# Germination Time and Temperature Modulate Bioactive Compound Accumulation in Black Rice (*Oryza sativa* L.) and Optimize Functional Water-Soluble Extract Production

**DOI:** 10.3390/plants15182748

**Published:** 2026-09-08

**Authors:** Larissa Karla de Jesus, Lara Tatiane Geremias Ferreira Brites, Alicia Lavado-Cruz, Irene Andressa, Georgia Ane Raquel Sehn, Ester Wickert, Nathalia de Andrade Neves, Marcio Schmiele

**Affiliations:** 1 Laboratório Integrado de Cereais e Lipídeos (LICEL), Instituto de Ciência e Tecnologia (ICT), Universidade Federal dos Vales do Jequitinhonha e Mucuri (UFVJM), Diamantina 39100-000, Brazil; larissa.karla@ufvjm.edu.br; 2 Departamento de Engenharia Agronômica, Centro Universitário de Araras Dr. Edmundo Ulson, Araras 13603-112, Brazil; larabrites@hotmail.com; 3 Departamento de Engenharia e Tecnologia de Alimentos, Faculdade de Engenharia de Alimentos, Universidade Estadual de Campinas (UNICAMP), Campinas 13083-862, Brazil; a298408@dac.unicamp.br; 4 Departamento de Tecnologia de Alimentos, Universidade Federal de Viçosa, Viçosa 36570-900, Brazil; irene.andressa@ufv.br; 5 Centro de Educação Superior do Oeste, Departamento de Engenharia de Alimentos e Engenharia Química, Universidade do Estado de Santa Catarina, Pinhalzinho 89870-000, Brazil; georgia.sehn@udesc.br; 6 Estação Experimental de Itajaí, Empresa de Pesquisa Agropecuária e Extensão Rural de Santa Catarina (EPAGRI), Itajaí 88318-112, Brazil; esterwickert@epagri.sc.gov.br; 7 Laboratório de Pesquisa em Bioativos de Plantas (FITOLAB), Departamento de Agronomia, Faculdade de Ciências Agrárias (FCA), Universidade Federal dos Vales do Jequitinhonha e Mucuri (UFVJM), Diamantina 39100-000, Brazil; nathalia.neves@ufvjm.edu.br

**Keywords:** GABA, phenylpropanoid metabolism, phytochemicals, response surface methodology, seed physiology

## Abstract

Seed germination induces profound physiological and metabolic changes that promote the synthesis and accumulation of bioactive metabolites in cereal grains. However, the combined effects of germination time and temperature on these responses in black rice (*Oryza sativa* L.) remain poorly understood. This study investigated how controlled germination modulates the accumulation of GABA (γ-aminobutyric acid) and phenolic metabolites and identified optimal conditions for obtaining bioactive-rich water-soluble extracts. A central composite design was applied by varying germination time (24–96 h) and temperature (14–32 °C). Germinated flours and their corresponding water-soluble extracts were analyzed for GABA, total soluble phenolics, flavonoids, anthocyanins, proanthocyanidins, and oxygen radical absorbance capacity (ORAC). Response surface methodology, desirability analysis, principal component analysis, heatmap, and partial least squares discriminant analysis were used to characterize metabolic responses and optimize germination conditions. Germination significantly altered metabolite accumulation, with higher GABA levels observed at lower temperatures, whereas phenolic metabolites showed distinct responses to the interaction between time and temperature. Multivariate analyses revealed clear metabolic differentiation among germination treatments. Multi-response optimization identified 27 h and 19.7 °C as the optimal germination conditions. These findings demonstrate that controlled germination modulates the bioactive profile of black rice, enhancing phytochemical accumulation and supporting the production of bioactive-rich water-soluble extracts.

## 1. Introduction

Rice (*Oryza sativa* L.) is one of the world’s most important staple crops, providing more than 20% of global dietary energy intake and serving as a primary food source for over half of the world’s population, making it essential for global food security [1]. Beyond conventional white rice, the species exhibits remarkable genetic diversity, giving rise to pigmented varieties such as black, red, and purple rice, which differ in grain color due to the accumulation of secondary metabolites in the outer layers of the grain [2,3]. Among these, black rice has attracted growing scientific and commercial interest due to its distinctive nutritional composition and diverse phytochemical profile. Compared with polished white rice, black rice generally provides higher levels of dietary fiber and bioactive constituents and has been reported to contain approximately 21.8% amylose and 68.1% starch, although its composition varies according to cultivar and growing conditions [3,4]. Its pigmented outer layers are particularly rich in phenolic compounds and anthocyanins, predominantly cyanidin-3-glucoside and peonidin-3-glucoside [5], as well as other bioactive constituents such as γ-oryzanol, tocopherols, tocotrienols, phytosterols, GABA, minerals, and phytic acid [6,7]. This complex phytochemical composition contributes substantially to the high antioxidant capacity of black rice and has been associated with anti-inflammatory and other health-promoting properties [6,8]. Despite its nutritional and functional potential, black rice remains comparatively underutilized because of its relatively low productivity and limited adoption of improved cultivation practices, highlighting the need for strategies that increase its value-added utilization and exploit its bioactive potential [9].

In this context, germination stands out as a promising biotechnological strategy to enhance the nutritional and functional value of black rice, as it promotes intense metabolic reprogramming capable of modifying the concentration, distribution, and bioavailability of different classes of bioactive compounds. During this process, enzymatic activation and mobilization of grain reserves may favor both the synthesis of new metabolites and the release of compounds previously associated with cellular structures, resulting in changes in the levels of GABA, phenolic acids, flavonoids, anthocyanins, proanthocyanidins, γ-oryzanol, and other bioactive constituents [10,11]. In particular, activation of the phenylpropanoid pathway and increased phenylalanine ammonia-lyase (PAL) activity may contribute to changes in the profile of phenolic acids and flavonoids, whereas glutamate decarboxylase (GAD) activity promotes the conversion of glutamate into GABA, leading to its accumulation during germination [12,13]. Recent evidence has demonstrated that germination of different rice varieties can markedly increase GABA levels while simultaneously enhancing the concentrations of certain phenolic acids, including compounds such as protocatechuic acid, in addition to modifying other bioactive components associated with antioxidant capacity [10,14,15]. In pigmented rice, this response is particularly relevant due to the presence of a complex phytochemical matrix characterized by anthocyanins, phenolic acids, and flavonoids, which contribute to its high antioxidant capacity [16]. However, germination does not necessarily result in a uniform increase in all these compounds, as simultaneous processes of synthesis, release, enzymatic transformation, oxidation, and degradation may occur throughout seed development. Thus, the final bioactive compound profile results from the dynamic balance among these metabolic pathways and is strongly influenced by germination conditions, particularly time, temperature, and water availability [17,18,19].

In germinated black rice, substantial phytochemical diversity has been reported, particularly within the phenolic fraction. Rashid and colleagues [20] identified several phenolic acids, including gallic, chlorogenic, ellagic, ferulic, hydroxybenzoic, isoferulic, p-coumaric, protocatechuic, sinapic, and vanillic acids. The same authors also reported flavonoids such as kaempferol, quercetin, and rutin, together with several anthocyanins, including cyanidin-3-galactoside, cyanidin-3-glucoside, cyanidin-3,5-diglucoside, cyanidin-3-rutinoside, and malvidin-3-galactoside. In parallel with phenolic metabolism, germination also promotes changes in other nutritionally relevant constituents, including free amino acids, minerals, and γ-aminobutyric acid (GABA), further contributing to the functional quality of germinated black rice [21]. Among these responses, GABA accumulation is well documented, with concentrations reported to increase from 40.73 to 258.35 mg kg^−1^ after 48 h of germination [19]. Overall, the magnitude and direction of germination-induced changes in both phenolic and non-phenolic bioactive compounds are strongly dependent on processing conditions. Germination regimes around 28–30 °C and 30–48 h have frequently been reported as favorable for black rice, although optimal conditions vary according to the targeted bioactive compound, cultivar, moisture level, and pretreatment conditions [22,23,24].

From a technological perspective, the recovery of water-soluble bioactive compounds from germinated grains is particularly relevant for the development of functional food ingredients [25,26]. Although organic solvents such as ethanol, methanol, and acetone are widely employed for analytical extraction of phytochemicals, water represents an attractive alternative for food applications because of its safety, environmental compatibility, and ability to selectively recover hydrophilic constituents [27,28]. In germinated cereals, such aqueous extracts may constitute promising ingredients for incorporation into functional beverages and other health-oriented food products [29]. Therefore, understanding how germination modulates not only the bioactive composition retained in the grain matrix but also the fraction that becomes recoverable in water is relevant for translating germination-induced metabolic changes into potential food applications.

Despite these advances, the combined effects of germination time and temperature on the bioactive profile of black rice remain insufficiently understood. Previous studies have often focused on individual compounds or employed elicitors and chemical pretreatments, while the simultaneous response of different phenolic classes, GABA, and antioxidant capacity has received limited attention. Furthermore, little is known about how these changes are reflected in both the grain matrix and its water-soluble fraction. Therefore, this study applied response surface methodology and multivariate analysis to evaluate the effects of germination time and temperature on GABA, total soluble phenolics, flavonoids, anthocyanins, proanthocyanidins, and antioxidant capacity in black rice flour and water-soluble extracts, and to identify conditions that maximize their overall bioactive potential.

## 2. Results

### 2.1. Proximate Composition of Black Rice Flour

Black rice flour exhibited a moisture content of 11.75 ± 0.10%. The flour contained 3.39 ± 0.29% lipids, 1.11% ash (<0.01), 13.10 ± 0.14% protein, and 70.65 ± 0.34% total carbohydrates, calculated by difference. Carbohydrates were the predominant component, followed by protein, lipids, and ash.

### 2.2. GABA Accumulation as an Indicator of Germination Efficiency

The GABA content varied markedly among the germination treatments (Table 1), confirming that germination conditions strongly influenced its accumulation in black rice. GABA was not detected in either the control or macerated samples, indicating that water absorption alone was insufficient to promote detectable GABA accumulation under the evaluated conditions. Among the evaluated treatments, GABA content varied markedly, ranging from 8.01 mg·100 g^−1^ to 21.02 mg·100 g^−1^, with the highest concentration recorded in treatment 7, corresponding to germination at 14 °C for 60 h.

**Figure 1:**
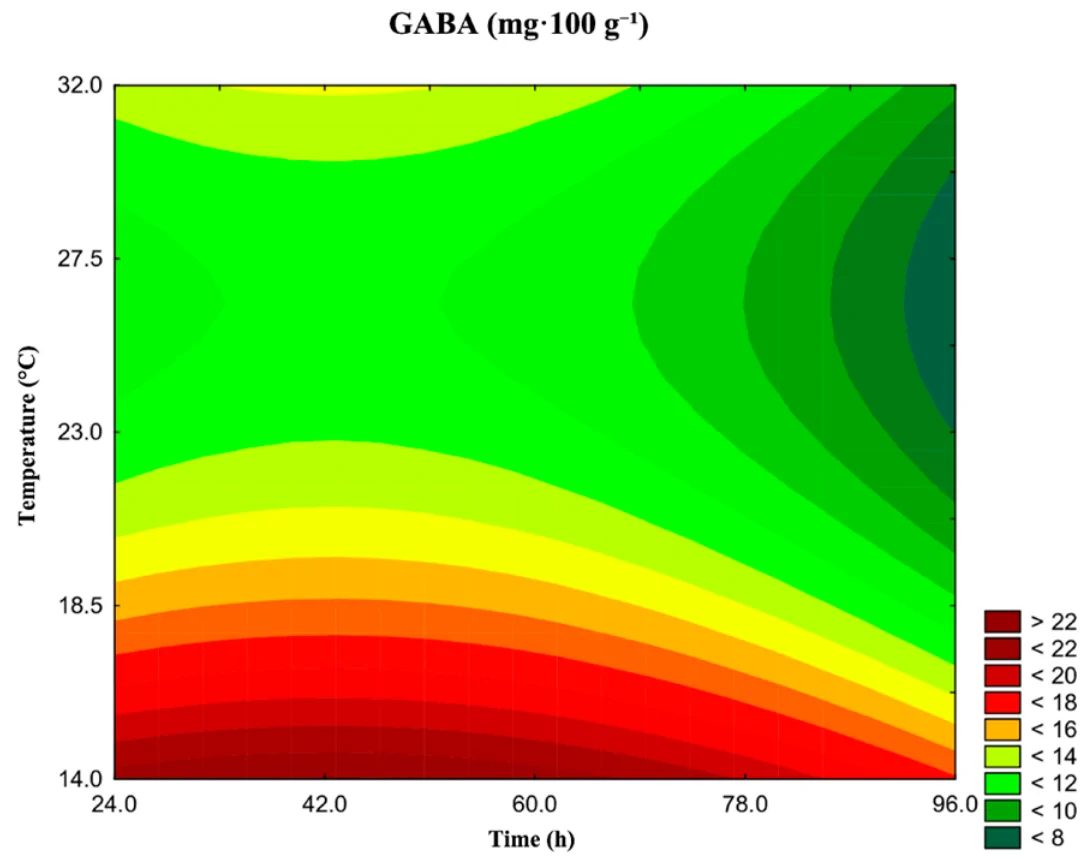
Contour plot for γ-aminobutyric acid (GABA) content as a function of germination time and temperature.

The fitted quadratic model adequately described GABA accumulation during black rice germination, explaining 95.55% of the experimental variability (R^2^ = 0.9555). Analysis of variance confirmed that the model was highly significant (F = 9.14, *p* < 0.001), indicating that the selected second-order polynomial satisfactorily represented the experimental data. Consequently, the model was considered suitable for predicting GABA accumulation within the evaluated experimental region and for generating the response surface and contour plots (Equation (1) and Figure 1).

(1) $$\mathrm{GABA}\;(mg\cdot 100\;g^{-1})=12.37-1.54x_{1}-2.80x_{2}-1.13x_{1}^{2}+2.63x_{2}^{2}$$

where $x_{1}$ and $x_{2}$ are the coded values of the independent variable’s germination time and germination temperature, respectively, and $x_{1}^{2}$ and $x_{2}^{2}$ are the corresponding quadratic terms of the coded variables. GABA, γ-aminobutyric acid.

Both germination time and temperature significantly influenced GABA accumulation. Germination time showed significant negative linear (*β*_1_ = −1.54, *p* = 0.003) and quadratic (*β*_11_ = −1.13, *p* = 0.023) effects, indicating that extending the germination period progressively reduced GABA accumulation. This response suggests that prolonged germination may promote the subsequent metabolism of GABA through the GABA shunt as seed metabolic activity increases. Temperature also exerted a significant effect, with a negative linear coefficient (*β*_2_ = −2.80, *p* < 0.001), demonstrating that increasing temperature reduced GABA accumulation. However, the positive quadratic effect (*β*_22_ = 2.63, *p* < 0.001) indicates a curvilinear response, suggesting that the inhibitory effect of temperature was partially attenuated within specific regions of the experimental domain.

### 2.3. Effect of Germination Conditions on Bioactive Compounds and Antioxidant Capacity of Black Rice Flours and Their Soluble Extracts

The contents of total soluble phenolic compounds, total flavonoids, total anthocyanins, total proanthocyanidins, and oxygen radical absorbance capacity (ORAC) determined in black rice flours and their corresponding water-soluble extracts obtained under the different germination conditions are presented in Table 2. Distinct responses were observed among the germination treatments in both sample matrices, demonstrating that germination time and temperature differentially influenced the accumulation, extractability, and antioxidant potential of the evaluated bioactive compounds.

#### 2.3.1. Total Soluble Phenolic Compounds (TSPC)

The total soluble phenolic compound (TSPC) content of germinated black rice flours ranged from 1059.01 to 1294.51 mg GAE·100 g^−1^. The highest value was observed in treatment 2 (1294.51 ± 3.10 mg GAE·100 g^−1^), obtained after 85.5 h of germination at 16.6 °C, whereas treatment 5, conducted for 24 h at 23 °C, exhibited the lowest content (1059.01 ± 46.43 mg GAE·100 g^−1^). The control and soaked samples contained 1172.93 ± 38.13 and 1155.56 ± 47.36 mg GAE·100 g^−1^, respectively, with no significant effect of soaking on TSPC (p=0.491). The fitted model explained 49.48% of the experimental variability but was not statistically significant (p=0.418), indicating that germination time and temperature did not produce a consistent response pattern within the experimental domain. Therefore, the differences observed among treatments were interpreted descriptively rather than through the fitted model.

**Figure 2:**
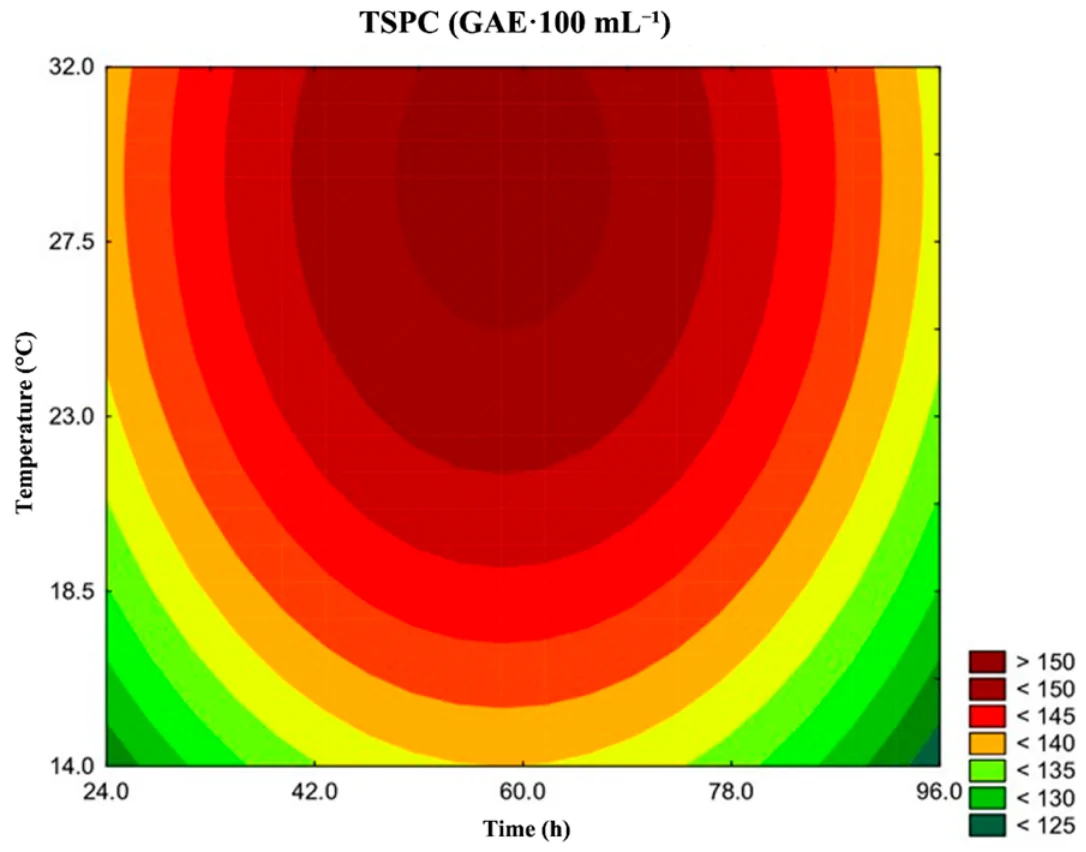
Contour plot for total soluble phenolic content (TSPC) as a function of germination time and temperature in soluble extracts of germinated black rice flour.

In the soluble extracts, TSPCs ranged from 129.36 to 149.37 mg GAE·100 mL^−1^. The lowest concentration was recorded in treatment 6 (129.36 ± 7.68 mg GAE·100 mL^−1^), while treatment 12 exhibited the highest value among the germinated samples (149.37 ± 5.85 mg GAE·100 mL^−1^). The mathematical model was significant (p=0.007) and explained 83.93% of the experimental variability, demonstrating an adequate fit for describing the effects of germination time and temperature. The linear temperature term exerted a positive effect on TSPC content ($\beta_{2}=4.60$, p<0.001; Equation (2)). Accordingly, the contour plot indicated that intermediate germination times combined with higher temperatures favored higher TSPC levels in the water-soluble extracts (Figure 2).

(2) $$\mathrm{TSPC}\;(\mathrm{mg}\;\mathrm{GAE}\cdot 100\;{\mathrm{mL}}^{-1})=148.68-0.90x_{1}+4.60x_{2}-6.63x_{1}^{2}-2.38x_{2}^{2}$$

where $x_{1}$ and $x_{2}$ represent the coded values of germination time and germination temperature, respectively, while $x_{1}^{2}$ and $x_{2}^{2}$ represent their corresponding quadratic terms. GAE, gallic acid equivalents.

#### 2.3.2. Total Flavonoid Content (TFC)

The total flavonoid content (TFC) of germinated black rice flours ranged from 375.02 to 834.18 mg QE·100 g^−1^. Soaking reduced TFC from 672.02 ± 112.33 mg QE·100 g^−1^ in the control flour to 471.26 ± 152.51 mg QE·100 g^−1^ in the soaked sample, corresponding to a significant loss of 29.87% (p=0.019). Among the germinated samples, the highest TFC was recorded in treatment 5 (834.18 ± 267.56 mg QE·100 g^−1^), followed by Treatments 3 (816.22 ± 112.84 mg QE·100 g^−1^), 7 (729.72 ± 205.05 mg QE·100 g^−1^), and 1 (716.74 ± 117.84 mg QE·100 g^−1^). In contrast, treatments 4 and 8 showed the lowest values, with 375.02 ± 49.49 and 410.25 ± 172.00 mg QE·100 g^−1^, respectively.

**Figure 3:**
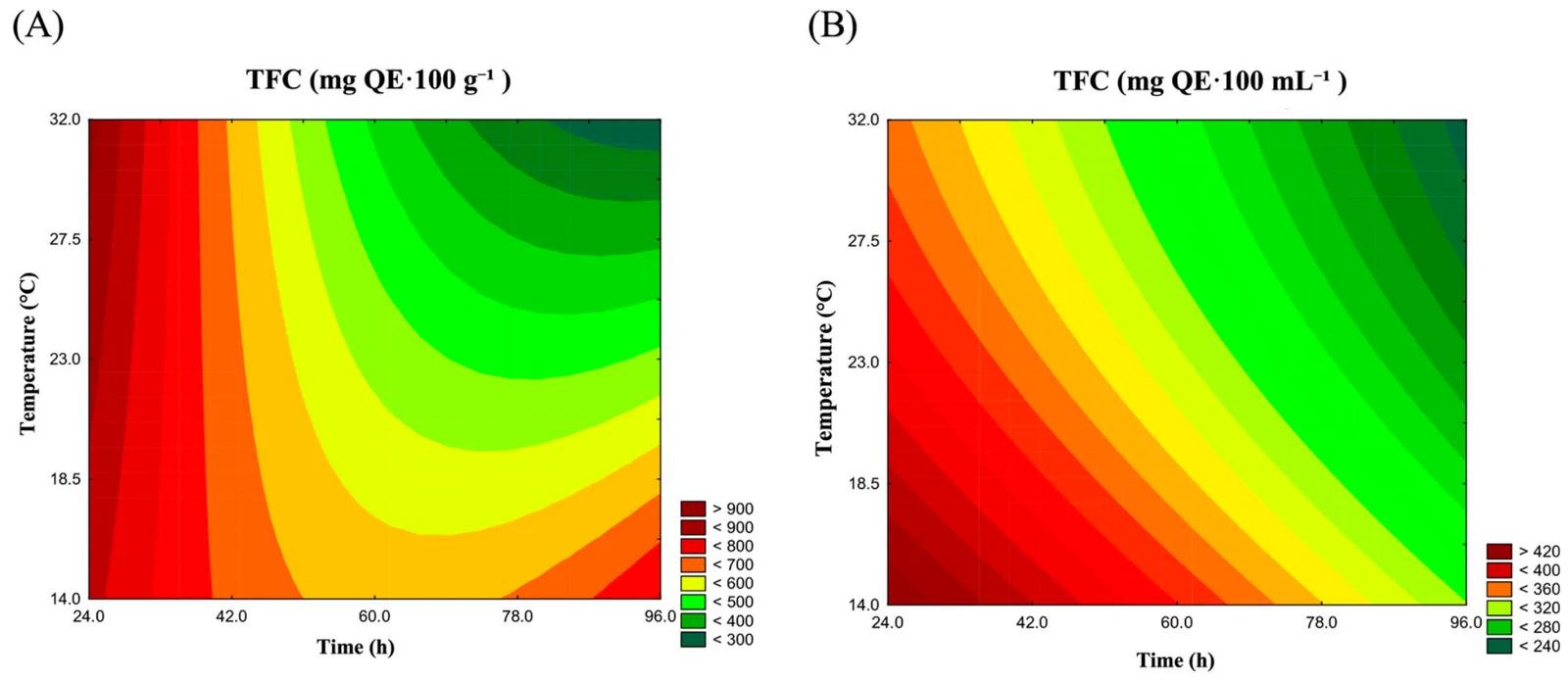
Contour plots for total flavonoid content as a function of germination time and temperature in (**A**) black rice flours and (**B**) their corresponding soluble extracts.

The fitted mathematical model was significant (p=0.005) and explained 84.93% of the experimental variability ($R^{2}=0.8493$; $F_{calc}/F_{tab}=2.39$). The quadratic term of germination time exerted a significant positive effect on TFC ($\beta_{11}=77.93$, p=0.034; Equation (3)), confirming a nonlinear response to germination duration. According to the contour plot (Figure 3A), higher flavonoid contents were favored by shorter germination periods over a relatively broad temperature range, as well as by longer germination periods combined with lower temperatures.

(3) $$\mathrm{TFC}\;(\mathrm{mg}\;\mathrm{QE}\cdot 100\;g^{-1})=148.68-0.90x_{1}+4.60x_{2}-6.63x_{1}^{2}-2.38x_{2}^{2}$$

where $x_{1}$ and $x_{2}$ are the coded values of the independent variable’s germination time and germination temperature, respectively, while $x_{1}^{2}$ and $x_{2}^{2}$ represent their corresponding quadratic terms. QE, quercetin equivalents.

In the soluble extracts, TFC ranged from 249.83 to 413.41 mg QE·100 mL^−1^. Treatment 1 exhibited the highest concentration (413.41 ± 46.33 mg QE·100 mL^−1^), followed by treatments 7 (362.68 ± 31.50 mg QE·100 mL^−1^), 3 (362.29 ± 18.32 mg QE·100 mL^−1^), and 5 (360.11 ± 18.89 mg QE·100 mL^−1^). These treatments also exceeded the control extract, which contained 302.89 ± 25.46 mg QE·100 mL^−1^. The lowest value was recorded in treatment 4 (249.83 ± 27.88 mg QE·100 mL^−1^).

The model fitted to the soluble extracts was statistically significant (p<0.001) and explained 93.46% of the experimental variability ($R^{2}=0.9346$; $F_{calc}/F_{tab}=9.37$). Germination time ($\beta_{1}=-42.41$, p<0.001) and temperature ($\beta_{2}=-26.80$, p<0.001) exerted negative linear effects on TFC, whereas the positive quadratic effect of temperature ($\beta_{22}=5.12$, p=0.0133) indicated curvature in the response (Equation (4)). The contour plot therefore showed that shorter germination periods, particularly when combined with mild temperatures, favored flavonoid recovery in the soluble extracts (Figure 3B).

(4) $$\mathrm{TFC}\;(\mathrm{mg}\;\mathrm{QE}\cdot 100\;{\mathrm{mL}}^{-1})=322.57-42.41x_{1}-26.80x_{2}+5.12x_{2}^{2}$$

where $x_{1}$ and $x_{2}$ are the coded values of the independent variable’s germination time and germination temperature, respectively, and $x_{2}^{2}$ is the quadratic term of the coded germination temperature variable. QE, quercetin equivalents.

#### 2.3.3. Total Anthocyanin Content (TAC)

The total anthocyanin (TAC) content of black rice flour was significantly affected by the germination conditions. The highest values were observed in treatments 1, 5, and 7, reaching 4642.81, 4538.73, and 4440.38 mg C3G·100 g^−1^, respectively. These treatments involved short to intermediate germination times (24–60 h) and low to moderate temperatures (14–23 °C), indicating that these conditions favored anthocyanin retention in the solid matrix.

**Figure 4:**
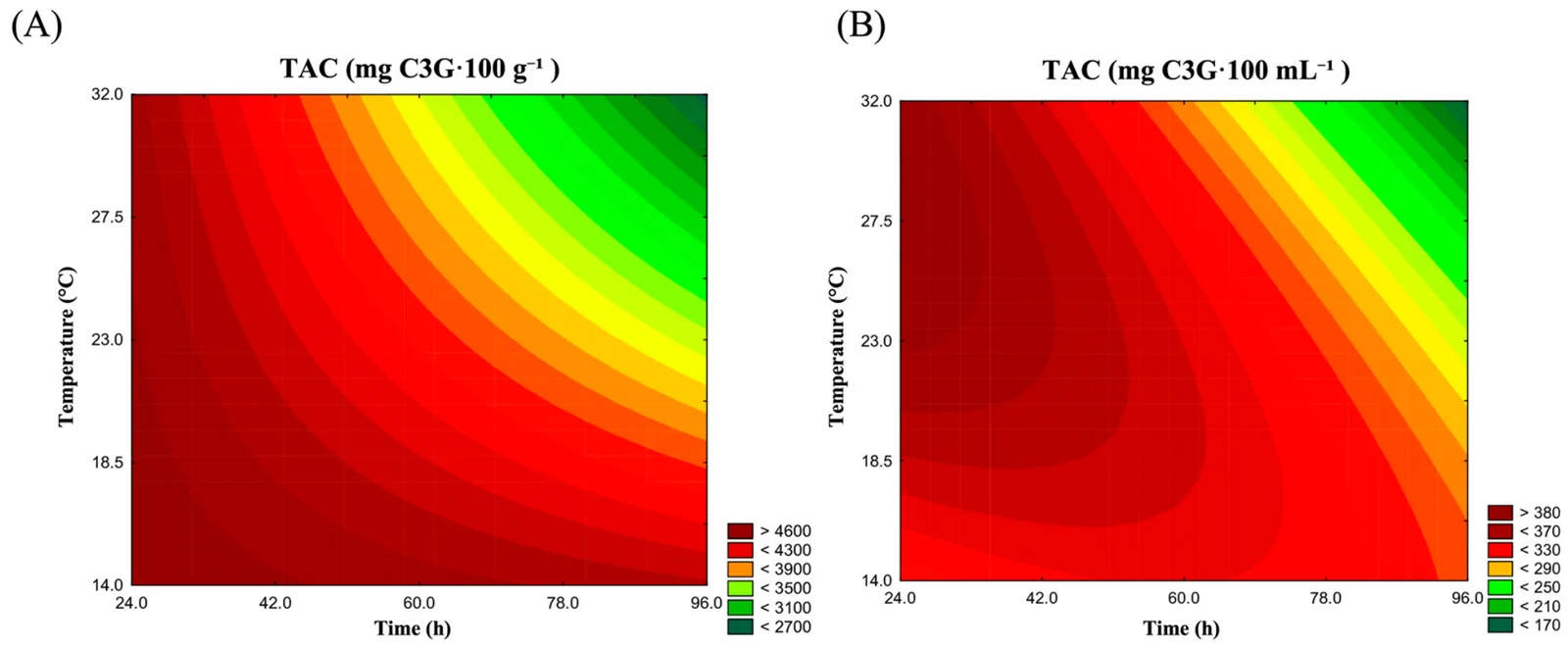
Contour plots for total anthocyanin content as a function of germination time and temperature in (**A**) black rice flours and (**B**) their corresponding soluble extracts.

Soaking reduced the anthocyanin content by 24.92% compared with the untreated control (*p* < 0.001). ANOVA indicated that the mathematical model fitted to the flour data was statistically significant and explained 94.97% of the observed variability (R^2^ = 0.9497; $F_{calc}/F_{tab\left(0.10\right)}=12.37$; *p* < 0.001). Therefore, Equation (5) and the corresponding contour plot (Figure 4A) adequately described the response within the experimental domain.

(5) $$\mathrm{TAC}\;(\mathrm{mg}\;C3G\cdot 100\;g^{-1})=4077.06-383.74x_{1}-336.05x_{2}-205.57x_{1}x_{2}$$

where $x_{1}$ and $x_{2}$ are the coded values of the independent variable’s germination time and germination temperature, respectively, and $x_{1}x_{2}$ represents the interaction between germination time and temperature. CEG, cyanidin-3-glucoside equivalents.

Germination time and temperature exerted significant negative linear effects on anthocyanin content, with coefficients of β_1_ = −383.74 (*p* < 0.001) and β_2_ = −336.05 (*p* < 0.001), respectively. A significant negative interaction between time and temperature was also observed (β_12_ = −205.57; *p* = 0.0097). The contour plot showed that anthocyanin retention was favored at shorter germination times and lower to moderate temperatures, whereas the simultaneous increase in both variables intensified anthocyanin losses.

In the soluble extracts, total anthocyanin content ranged from 226.72 to 373.04 mg C3G·100 mL^−1^. The highest value was obtained for treatment 5 and exceeded that of the control extract, which contained 339.63 mg C3G·100 mL^−1^. This result indicates that selected germination conditions increased the amount of extractable anthocyanins in the aqueous extract.

The model fitted to the soluble-extract data was statistically significant and explained 88.66% of the response variability (R^2^ = 0.8866; $F_{calc}=9.38$; $F_{tab}=4.39$; $F_{calc}/F_{tab\left(0.10\right)}=2.14$; *p* = 0.008). Equation (6) and its corresponding contour plot (Figure 4B) adequately represented the effects of germination time and temperature on anthocyanin content in the soluble extracts.

(6) $$\mathrm{TAC}\;(\mathrm{mg}\;C3G\cdot 100\;{\mathrm{mL}}^{-1})=339.76-38.82x_{1}-13.29x_{2}-25.95x_{1}x_{2}-11.22x_{1}^{2}-13.90x_{2}^{2}$$

where $x_{1}$ and $x_{2}$ are the coded values of the independent variable’s germination time and germination temperature, respectively, while $x_{1}^{2}$ and $x_{2}^{2}$ represent their corresponding quadratic terms, and $x_{1}x_{2}$ represents the interaction between germination time and temperature. C3G, cyanidin-3-glucoside equivalents.

Germination time showed a significant negative linear effect (β_1_ = −38.82; *p* < 0.001) and a significant negative quadratic effect (β_11_ = −11.22; *p* = 0.049). Temperature also exhibited negative linear (β_2_ = −13.29; *p* = 0.024) and quadratic effects (β_22_ = −13.90; *p* = 0.028). Furthermore, the interaction between germination time and temperature was negative and significant (β_12_ = −25.95; *p* = 0.0097), indicating that prolonged germination combined with higher temperatures caused the greatest reductions in extractable anthocyanins.

#### 2.3.4. Total Proanthocyanidin Content (TPAC)

The total proanthocyanidin content (TPAC) of black rice flours varied from 139.57 to 304.17 mg CE·100 g^−1^. The highest concentration was observed in treatment 5 (304.17 mg CE·100 g^−1^), corresponding to germination for 24 h at 23 °C, whereas treatment 4 showed the lowest value (139.57 mg CE·100 g^−1^). These results indicate that short germination periods under moderate-temperature conditions favored proanthocyanidin retention in the flour.

**Figure 5:**
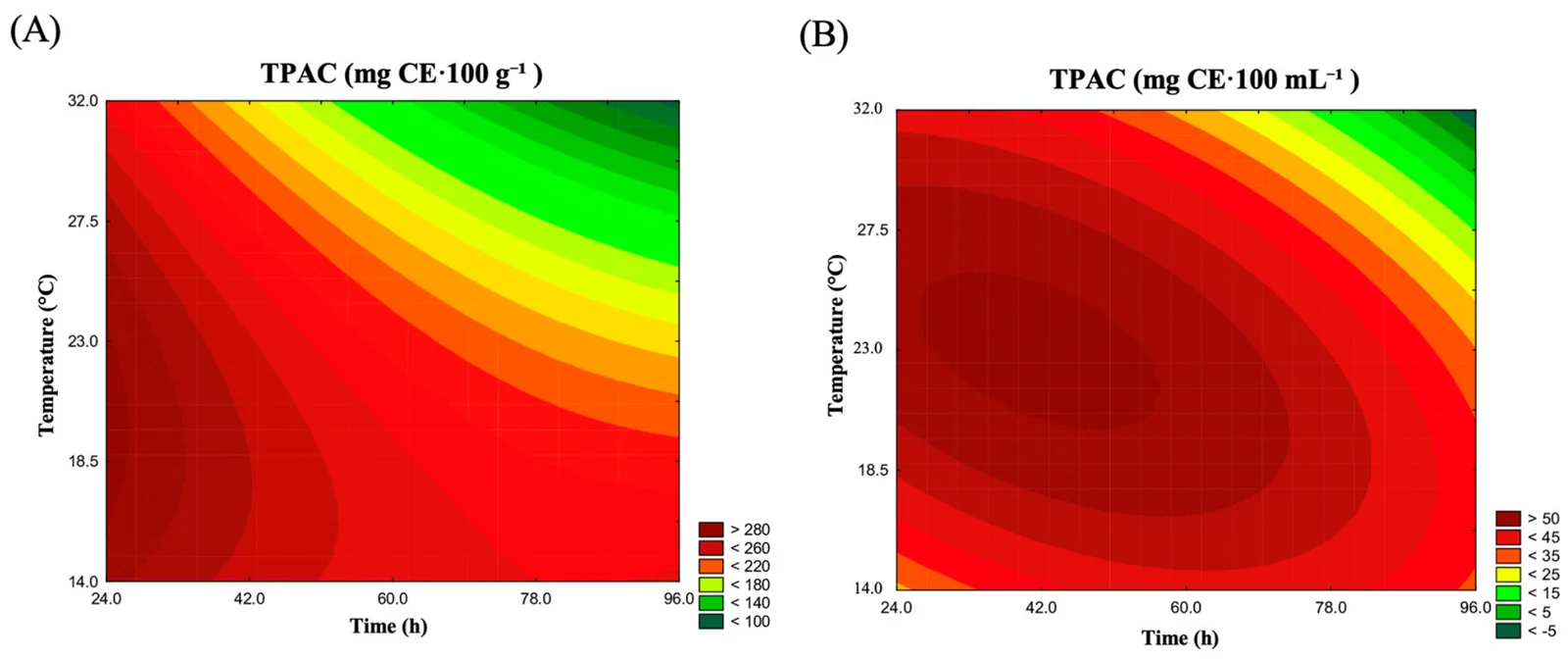
Contour plots for total proanthocyanidin content as a function of germination time and temperature in (**A**) black rice flours and (**B**) their corresponding soluble extracts.

Soaking reduced the proanthocyanidin content by 44.42% compared with the untreated control (*p* < 0.001). ANOVA showed that the model fitted to the flour data was statistically significant and explained 92.47% of the experimental variability (R^2^ = 0.9247; $F_{calc}/F_{tab\left(0.10\right)}=3.36$; *p* = 0.003). Therefore, Equation (7) and the corresponding contour plot (Figure 5A) adequately described the effects of germination time and temperature within the experimental domain.

(7) $$\mathrm{TPAC}\;(\mathrm{mg}\;\mathrm{CE}\cdot 100\;g^{-1})=226.30-30.71x_{1}-32.30x_{2}-13.78x_{1}x_{2}+6.12x_{1}^{2}-13.52x_{2}^{2}$$

where $x_{1}$ and $x_{2}$ are the coded values of the independent variable’s germination time and germination temperature, respectively, while $x_{1}^{2}$ and $x_{2}^{2}$ represent their corresponding quadratic terms, and $x_{1}x_{2}$ represent the interaction between germination time and temperature. CE, catechin equivalents.

Germination time exerted a significant negative linear effect on proanthocyanidin content (β_1_ = −30.71; *p* < 0.001). Its positive quadratic term was also significant (β_11_ = 6.12; *p* = 0.046), demonstrating curvature in the response and indicating that the effect of time was not strictly linear. Temperature presented a significant negative linear effect (β_2_ = −32.30; *p* < 0.001), together with a significant negative quadratic effect (β_22_ = −13.52; *p* = 0.005). The interaction between germination time and temperature was also negative and significant (β_12_ = −13.78; *p* = 0.010). The contour plot indicated that proanthocyanidin retention was favored by shorter germination periods and lower-to-moderate temperatures, whereas prolonged germination combined with higher temperatures intensified the reduction in these compounds.

In the corresponding soluble extracts, total proanthocyanidin content ranged from 13.51 to 59.05 mg CE·100 mL^−1^. Treatment 5 presented the highest concentration, whereas treatment 4 showed the lowest value, following the same general pattern observed in the flours. The control extract contained 63.27 mg CE·100 mL^−1^, which was numerically higher than the values obtained for the germinated samples. Nevertheless, extracts from treatments 5 and 7 and from the central-point treatments retained concentrations above 50 mg CE·100 mL^−1^.

The model fitted to the soluble-extract data was statistically significant and explained 83.16% of the observed variability (R^2^ = 0.8316; $F_{calc}/F_{tab\left(0.10\right)}=1.35$; *p* = 0.026). Equation (8) and the corresponding contour plot (Figure 5B) therefore adequately represented the effects of the evaluated germination conditions on proanthocyanidin content in the soluble extracts.

(8) $$\mathrm{TPAC}\;(\mathrm{mg}\;\mathrm{CE}\cdot 100\;{\mathrm{mL}}^{-1})=53.58-8.37x_{1}-5.26x_{2}-7.22x_{1}x_{2}-5.82x_{1}^{2}-9.16x_{2}^{2}$$

where $x_{1}$ and $x_{2}$ are the coded values of the independent variables’ germination time and germination temperature, respectively, while $x_{1}^{2}$ and $x_{2}^{2}$ represent their corresponding quadratic terms, and $x_{1}x_{2}$ represents the interaction between germination time and temperature. CE, catechin equivalents.

Germination time showed significant negative linear (β_1_ = −8.37; *p* = 0.004) and quadratic effects (β_11_ = −5.82; *p* = 0.016). Temperature also exerted significant negative linear (β_2_ = −5.26; *p* = 0.016) and quadratic effects (β_22_ = −9.16; *p* = 0.005). Furthermore, the interaction between time and temperature was negative and significant (β_12_ = −7.22; *p* = 0.017), indicating that the simultaneous increase in both variables intensified the reduction in extractable proanthocyanidins.

#### 2.3.5. Oxygen Radical Absorbance Capacity (ORAC)

The oxygen radical absorbance capacity of germinated black rice flours ranged from 25.24 to 29.30 µmol TE·100 g^−1^. All germinated treatments showed numerically higher ORAC values than the untreated control flour, which presented 22.97 µmol TE·100 g^−1^. Treatment 2, corresponding to germination for 85.5 h at 16.6 °C, showed the highest antioxidant capacity.

The model fitted to the flour data explained 71.63% of the experimental variability (R^2^ = 0.7163); however, it was not statistically significant (*p* = 0.418). Therefore, the model was not considered suitable for predicting ORAC values within the experimental domain.

In the corresponding soluble extracts, ORAC values ranged from 2185.26 to 3306.57 µmol TE·100 mL^−1^. The highest value was obtained for treatment 4 (3306.57 µmol TE·100 mL^−1^), followed by treatments 8 (3030.23 µmol TE·100 mL^−1^) and 6 (3010.80 µmol TE·100 mL^−1^). The untreated control extract presented an intermediate value of 2851.54 µmol TE·100 mL^−1^, whereas the lowest ORAC values were observed in the central-point treatments.

**Figure 6:**
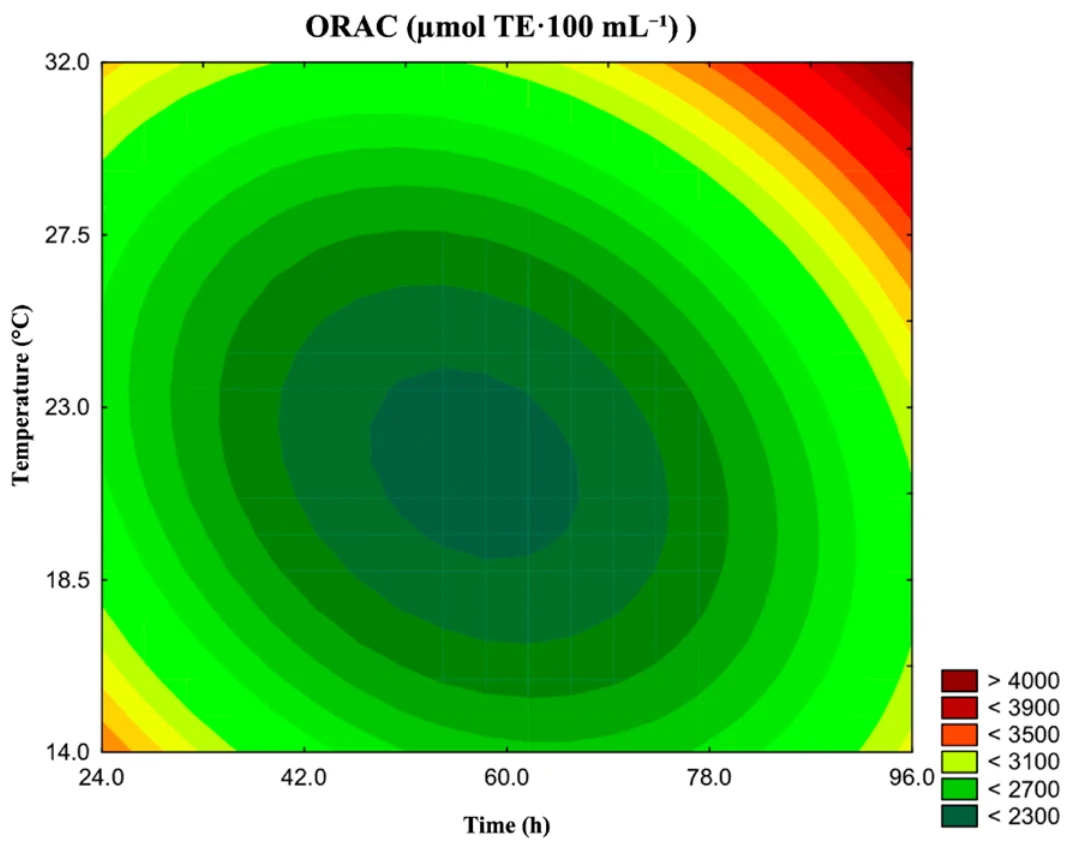
Contour plot for oxygen radical absorbance capacity (ORAC) as a function of germination time and temperature in soluble extracts of germinated black rice flour.

ANOVA showed that the model fitted to the soluble-extract data was statistically significant and explained 95.15% of the experimental variability (R^2^ = 0.9515; $F_{calc}/F_{tab\left(0.10\right)}=5.37$; *p* = 0.001). Equation (9) and the corresponding contour plot (Figure 6) therefore adequately described the effects of germination time and temperature on ORAC within the experimental domain.

(9) $$\mathrm{ORAC}\;(\mu \mathrm{mol}\;\mathrm{TE}\cdot 100\;{\mathrm{mL}}^{-1})=2280.26+107.62x_{1}+144.99x_{2}+142.09x_{1}x_{2}+322.88x_{1}^{2}+278.23x_{2}^{2}$$

where $x_{1}$ and $x_{2}$ are the coded values of the independent variable’s germination time and germination temperature, respectively, while $x_{1}^{2}$ and $x_{2}^{2}$ represent their corresponding quadratic terms, and $x_{1}x_{2}$ represent the interaction between germination time and temperature. TE, Trolox equivalents.

The quadratic effect of germination time was positive and significant (β_11_ = 322.87; *p* = 0.006), as was the quadratic effect of temperature (β_22_ = 278.23; *p* = 0.010). Temperature also exerted a significant positive linear effect (β_2_ = 144.99; *p* = 0.047). In contrast, the positive linear effect of germination time did not reach statistical significance at the 5% level (β_1_ = 107.62; *p* = 0.083). The interaction between time and temperature was also not significant (β_12_ = 142.09; *p* = 0.096), although it showed a positive tendency. The contour plot indicated that higher ORAC values were associated with combinations located toward the more intense regions of the experimental domain, whereas intermediate combinations produced lower responses.

### 2.4. Multivariate Analysis of Germination Responses

**Figure 7:**
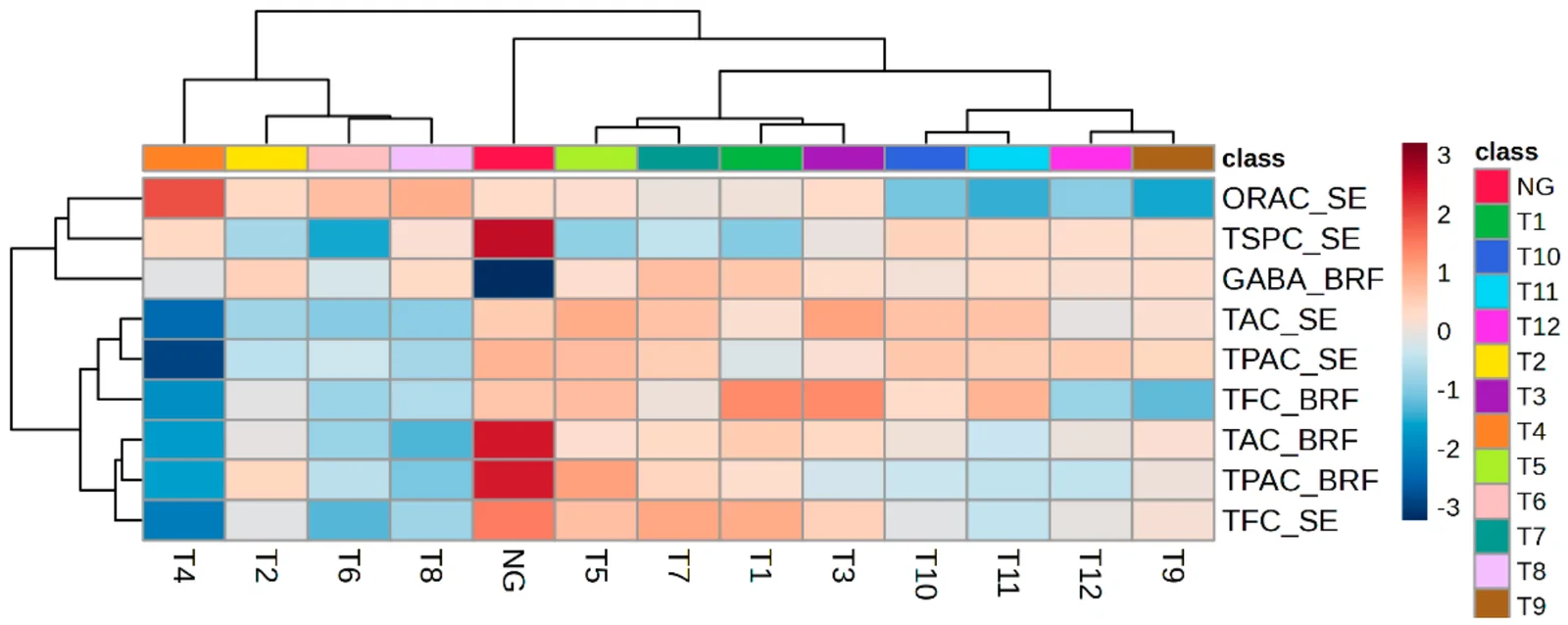
Hierarchical clustering heatmap showing the distribution of bioactive compounds in black rice samples subjected to different germination conditions. NG refers to the control treatment consisting of non-germinated black rice. T1–T12 correspond to the experimental treatments defined by the experimental design. ORAC, oxygen radical absorbance capacity; TSPC, total soluble phenolic compounds; GABA, γ-aminobutyric acid; TAC, total anthocyanin content; TPAC, total proanthocyanidin content; TFC, total flavonoid content; BRF, black rice flour; SE, soluble extract.

The hierarchical clustering heatmap (Figure 7) revealed distinct patterns in the bioactive composition of black rice samples, demonstrating that germination markedly altered their biochemical profiles. The untreated control formed an isolated cluster, clearly separated from all germinated treatments, indicating substantial differences in the overall composition of the evaluated bioactive compounds. The central-point treatments (9, 10, 11, and 12), corresponding to identical germination conditions (60 h and 23 °C), clustered closely together, demonstrating high experimental reproducibility under the selected processing conditions. Only minor differences in the abundance of some variables were observed within this group. Another cluster comprised treatments 2 (85.5 h, 16.6 °C), 4 (85.5 h, 29.4 °C), 6 (96 h, 23 °C), and 8 (60 h, 32 °C), indicating that these treatments shared similar multivariate profiles despite differences in germination temperature. In contrast, treatments 1, 3, 5, and 7 exhibited distinct distribution patterns, reflecting greater variability in the accumulation of bioactive compounds.

The heatmap also revealed marked differences in the distribution of individual response variables. GABA, total soluble phenolic compounds, anthocyanins, proanthocyanidins, flavonoids, and ORAC showed different accumulation patterns among the germination treatments, confirming that germination time and temperature differentially affected the evaluated bioactive compounds.

**Figure 8:**
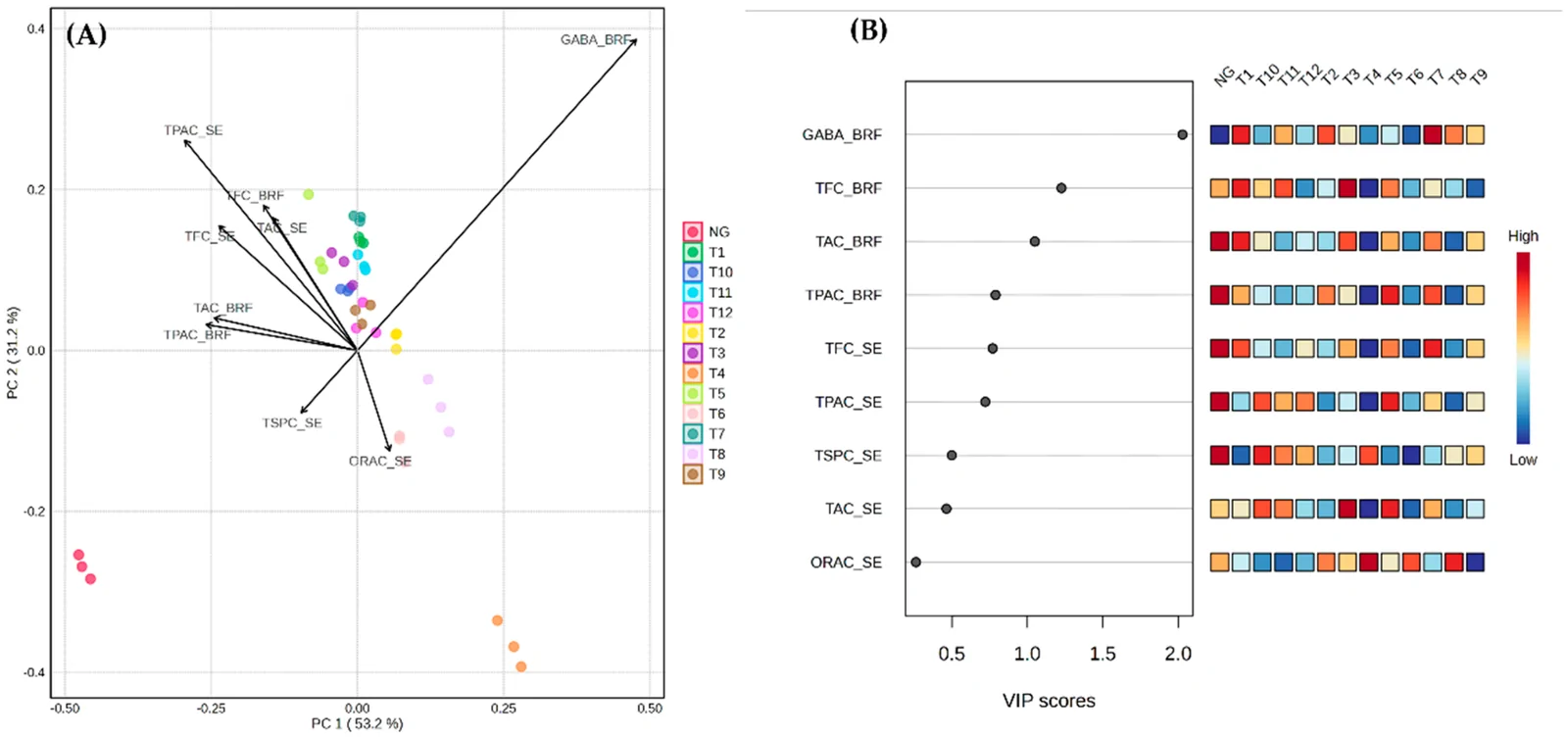
Principal component analysis (PCA) and partial least squares discriminant analysis (PLS-DA) of bioactive compounds in black rice samples subjected to different germination conditions: (**A**) PCA score and loading plots and (**B**) PLS-DA score plot with variable importance in projection (VIP) scores. NG refers to the control treatment consisting of non-germinated black rice. T1–T12 correspond to the experimental treatments defined by the experimental design. ORAC, oxygen radical absorbance capacity; TSPC, total soluble phenolic compounds; GABA, γ-aminobutyric acid; TAC, total anthocyanin content; TPAC, total proanthocyanidin content; TFC, total flavonoid content; BRF, black rice flour; SE, soluble extract.

Principal component analysis (PCA) and partial least squares discriminant analysis (PLS-DA) (Figure 8) were performed to investigate the multivariate relationships among germination treatments and to identify the bioactive compounds responsible for sample discrimination.

The PCA score plot (Figure 8A) explained 81.5% of the total variance through the first two principal components, indicating that the multivariate model effectively captured the major sources of metabolic variation among the samples. A clear separation of the untreated control from all germinated samples was observed, demonstrating that germination induced pronounced changes in the overall phytochemical composition of black rice. This discrimination indicates that germination produced coordinated changes in the measured bioactive compounds, resulting in a distinct phytochemical profile compared with the non-germinated control. Among the germinated treatments, treatment 7 was positioned separately along PC1 and was closely associated with GABA accumulation in the flour. Treatments 1 and 5 were clustered in the same region of the score plot and were associated with higher concentrations of anthocyanins, flavonoids, and proanthocyanidins in both flours and soluble extracts. In contrast, the central-point treatments (9–12) formed a compact cluster, confirming the high reproducibility of the germination process under identical experimental conditions. Variables related to antioxidant capacity (ORAC) and total soluble phenolic compounds in the extracts also contributed to treatment discrimination, particularly for treatments 3 and 4, which were positioned closer to these response variables in the loading plot.

The PLS-DA analysis (Figure 8B) further confirmed the separation among treatments and identified the variables contributing most strongly to class discrimination. Variable importance in projection (VIP) scores indicated that GABA content in the flour was the most influential variable in the model, followed by flavonoids and anthocyanins. These variables were primarily associated with treatments 7, 1, and 5, respectively, whereas variables measured in the soluble extracts, including total soluble phenolic compounds, ORAC, and anthocyanins, showed lower but still relevant contributions to the discrimination of treatments 3 and 4.

### 2.5. Numerical Optimization of Germination Condition

A numerical multi-response optimization was performed to determine the germination conditions that simultaneously maximized the statistically significant bioactive compounds described by predictive mathematical models (*p* < 0.10, F_calc_/F_tab_ > 1, and R^2^ > 0.80). Germination time and temperature were maintained within the experimental range, both assigned an importance level of 3, whereas all response variables were set to maximize with the highest importance (5). The optimization criteria and predicted responses are summarized in Table 3.

The optimization identified an optimal germination condition of 27 h at 19.7 °C, corresponding to an overall desirability of 70.81%. This solution reflects the best compromise among all responses, indicating that relatively short germination times combined with moderate temperatures favor the simultaneous accumulation of several bioactive compounds.

Model validation was carried out by comparing the predicted values with the experimental results obtained under the optimal germination conditions. According to the criterion proposed by [30], mathematical models presenting a relative deviation lower than 20% were considered satisfactorily validated. Under this criterion, the models for total flavonoids in the extract, total anthocyanins in both flour and extract, and proanthocyanidins in both flour and extract showed good predictive performance, with relative deviations ranging from 3.50% to 16.22%. In contrast, the models for GABA, total soluble phenolic compounds in the extract, total flavonoids in the flour, and ORAC of the extract exhibited larger deviations (>20%), indicating lower predictive accuracy under the optimized conditions.

## 3. Discussion

### 3.1. Reprogramming of Primary Metabolism and Physiological Activation of the Seed

The proximate composition of the black rice flour reflected a protein-rich matrix with characteristic modifications driven by processing and genetic factors. Moisture content remained below the maximum threshold of 13% required for safe storage, while lipid content (3.4–3.6%) aligned with values previously reported for black rice [31,32]. Notably, protein content was higher than ranges reported for black (8.84–10.10%) and other pigmented rice flours (7.44–8.46%) [33]. Conversely, ash and digestible carbohydrate levels were lower than those described for commercial cultivars [34,35]. While agronomic conditions and genotype significantly influence compositional variability [36], the reduced ash content is largely explained by the use of dehulled flour. Dehulling removes the outer bran layers where minerals, fiber, and specific lipids are concentrated [37], leaving an endosperm matrix enriched in protein reserves available for physiological mobilization.

This elevated protein baseline serves as the functional substrate during early seed hydration. Imbibition activates endogenous proteases that hydrolyze storage proteins, releasing free L-glutamate into the cellular pool [38]. This amino acid acts as the direct substrate for glutamate decarboxylase (GAD), which catalyzes the irreversible conversion of L-glutamate to gamma-aminobutyric acid (GABA) a primary marker of seed metabolic activation [39,40]. Extended germination provides the necessary timeframe for sustained proteolysis, continuous glutamate supply, and prolonged GAD activity [41,42]. However, cellular GABA accumulation is non-linear; it reflects a dynamic equilibrium between continuous synthesis and catabolism via GABA transaminase to feed the tricarboxylic acid (TCA) cycle for energy production [40,43].

Beyond primary energy mobilization, GABA synthesis functions as a protective metabolic response to environmental conditions. Moderate-to-low temperatures combined with hydration impose mild abiotic stress that stimulates GABA accumulation to regulate cytosolic pH, carbon/nitrogen balance, and redox homeostasis [44,45]. This metabolic activation highlights the physiological difference between simple hydration and active germination. Under the extraction and analytical conditions applied, GABA remained undetected in both the non-germinated control and soaked samples. Although unexpected relative to some literature values, repeated measurements confirmed this result, indicating that pre-germination GABA levels were below the analytical detection limit rather than entirely absent from the matrix. In contrast, active germination induced a pronounced metabolic shift, yielding consistent GABA accumulation ranging from 8.01 to 21.02 mg·100 g^−1^ and confirming GABA as a highly sensitive indicator of germination-driven primary reprogramming.

### 3.2. Dynamics of Phenylpropanoid Metabolism: Solubilization, De Novo Biosynthesis, and Degradation in the Solid Matrix and Extract

Seed germination fundamentally alters the phenolic profile of pigmented rice through a dynamic equilibrium between structural disassembly, de novo biosynthesis, physical leaching, and oxidative degradation [46,47]. Rather than a simple linear accumulation, the behavior of total soluble phenolic content (TSPC) during this process reflects these competing rate mechanisms. During hydration and early germination, endogenous cell wall-degrading enzymes and β-glucosidases disrupt interactions between phenolics and structural polysaccharides, enhancing the extractability of matrix-associated compounds [48]. Simultaneously, embryo activation triggers the phenylpropanoid pathway, promoting the synthesis of phenolic intermediates [49]. However, these synthetic gains are offset by concurrent leaching, polyphenol oxidase-mediated oxidation, and metabolic consumption during seedling development [50,51,52]. This non-linear dynamic, also observed by [53] in brown and red rice, explains why flour TSPC fluctuated, peaking under specific conditions (e.g., Treatment 2) but lacking a statistically significant predictive model across the entire experimental space.

Crucially, this metabolic reorganization directly dictates the solid-to-liquid partitioning during aqueous extraction. Although processing temperature positively influenced TSPC recovery within germinated soluble extracts, the ungerminated control extract maintained a slightly higher overall concentration. This indicates that germination modifies the structural distribution and binding states of phenolics rather than causing a net surge in total aqueous solubility. Phenolics released during germination can form insoluble complexes with storage proteins or altered cell wall polysaccharides, limiting their recovery in the aqueous phase [54].

In contrast to TSPC, flour flavonoid content (TFC) exhibited greater dynamic variability and a distinct recovery pattern. An initial decline following soaking aligned with the diffusion of extractable flavonoid fractions into the hydration medium. However, subsequent germination partially restored flour TFC, suggesting that metabolic activation, specifically the upregulation of the phenylpropanoid-flavonoid pathway and the enzymatic release of conjugated flavonoids, compensated for early leaching losses. This matrix transformation profoundly benefited the aqueous extracts: soluble extracts obtained under short-to-intermediate germination times displayed higher TFC values than the ungerminated control. Controlled germination effectively catalyzed the enzymatic conversion of cell wall-associated flavonoids into more polar, soluble forms, facilitating their transfer into the aqueous phase [55,56]. Prolonged germination or elevated temperatures reversed this advantage, promoting enzymatic degradation and polymerization.

Total anthocyanin content (TAC) demonstrated the highest sensitivity to these competing physicochemical forces. In the solid flour matrix, early germination induced a marked TAC decrease, a phenomenon widely corroborated in pigmented rice due to the high water solubility and pericarp localization of these pigments [57,58,59]. Imbibition facilitates external leaching, while simultaneously driving chemical degradation via pyrylium ring opening and subsequent chalcone formation [60]. Although germination selectively stimulates specific phenolic acids, anthocyanins generally experience a net loss in the solid matrix due to concurrent hydrolysis and endogenous oxidation [61].

Remarkably, despite this absolute depletion in the germinated flours, intermediate germination conditions yielded soluble extracts with higher anthocyanin concentrations than the non-germinated control. This apparent paradox is explained by matrix mechanics: germination-induced enzymatic modification of cell wall polysaccharides weakens pigment–matrix interactions, dramatically increasing anthocyanin accessibility during subsequent liquid extraction [62,63]. These structural shifts effectively break down cellular barriers, prioritizing extraction efficiency over total initial concentration. Naturally, this advantage is lost if processing is extended; elevated temperatures and prolonged germination times strictly accelerate thermal and oxidative pigment destruction.

Finally, total proanthocyanidins (TPAC) exhibited partitioning behavior mirroring that of anthocyanins. The initial marked decrease in flour TPAC following soaking reflects the loss of highly soluble oligomeric fractions and hydration-induced re-association with structural matrix components [58]. Because proanthocyanidins possess variable solubility dictated by their degree of polymerization, their dynamics represent a delicate equilibrium between the release of bound forms and concurrent oxidative polymerization. While TPAC levels in germinated soluble extracts remained generally lower than those in the ungerminated control, treatments conducted under moderate conditions retained substantial concentrations. Consistent with the TAC findings, partial cell wall breakdown during controlled germination facilitates the selective release of lower-molecular-weight proanthocyanidin oligomers into the aqueous phase without triggering extensive condensation [63].

Together, these results confirm that early-to-intermediate germination operates as an effective biological pretreatment. By precisely balancing structural softening against oxidative degradation, the extraction of specific functional flavonoids and thermosensitive anthocyanins can be maximized.

### 3.3. Mechanisms of Antioxidant Capacity and Phytochemical Synergy

The ORAC assay provided critical insights into the peroxyl radical-scavenging dynamics of both the solid flour matrix and the aqueous extracts. Consistent with established literature on pigmented rice, germination generally enhanced the baseline antioxidant capacity of the whole-grain flours [55,64]. However, the mathematical models revealed divergent behaviors between the solid and liquid phases. While the flour ORAC lacked the statistical significance required to map a continuous response surface, the soluble extracts exhibited a highly significant, U-shaped quadratic response. This non-linear dynamic dictates that liquid-phase antioxidant recovery is exquisitely sensitive to the physicochemical balance between enzymatic matrix liberation and thermal degradation during mashing.

A pivotal finding of this study is the partial decoupling of TSPC and TFC concentrations from total ORAC values. Higher total phenolic or flavonoid yields did not proportionally translate into maximized radical-scavenging capacity. This discrepancy underscores that overall antioxidant activity is governed by the specific phytochemical profile and molecular structures rather than sheer quantitative abundance [65]. The radical-quenching efficiency of phenolic compounds is strictly dictated by their degree of hydroxylation, glycosylation, and spatial conformation, structural features that are continuously altered by germination-induced enzymatic reactions and subsequent thermal hydrolysis [66].

This functional resilience is driven by two primary mechanisms: intermolecular synergy and the generation of non-phenolic antioxidants. First, diverse bioactive species, such as anthocyanins interacting with hydrophilic or lipophilic co-antioxidants, can amplify free-radical scavenging well beyond their additive individual contributions [67]. Second, the profound metabolic remodeling induced by germination generates a parallel suite of radical scavengers. The de novo synthesis of GABA, γ-oryzanol, and ascorbic acid, alongside the proteolytic release of low-molecular-weight bioactive peptides, provides alternative quenching mechanisms [68]. These non-phenolic metabolites act cooperatively with soluble phenolics to regenerate oxidized intermediates across different micro-environments within the extract [69], ensuring a robust and resilient antioxidant capacity in the final germinated product.

### 3.4. Multivariate Synthesis of Germination Profiles

The integrated multivariate evaluation, combining hierarchical clustering heatmaps, PCA, and PLS-DA, demonstrates that germination induces a profound, coordinated metabolic reprogramming in black rice rather than triggering uniform shifts across all compound classes [69,70]. Across all projection models, the untreated control was isolated in a distinct cluster, confirming that hydration and subsequent activation of primary and secondary metabolic pathways yield a biochemical profile fundamentally different from that of the raw grain, consistent with comparative metabolomic findings in other sprouted cereals [71]. Furthermore, the compact clustering of central-point treatments highlights the high experimental reproducibility of the process under standardized conditions, with minor variations reflecting the intrinsic biological heterogeneity of cereal seeds.

The spatial distribution of treatments in the PCA and PLS-DA score plots revealed that process variables dictate specific metabolic trajectories. Variable Importance in Projection (VIP) scores identified GABA content in the solid matrix, alongside extractable flavonoids and anthocyanins, as the primary discriminants driving class separation. This multivariate divergence perfectly encapsulates the physiological mechanisms operating within the grain. The marked VIP dominance of GABA (reaching 8.01–21.02 mg·100 g^−1^) reflects the rapid mobilization of storage reserves and activation of endogenous enzymes, such as glutamate decarboxylase (GAD), representing a dynamic balance between its de novo synthesis and utilization via the GABA shunt [38,42,43].

Simultaneously, the distinct clustering of phenolic responses mathematically visualizes the concurrent, yet divergent, mechanisms affecting phenylpropanoid metabolism. Moderate germination regimes clustered together because they favored the enzymatic release of conjugated flavonoids and activation of the phenylpropanoid pathway [13], effectively balancing metabolite synthesis against degradation. Conversely, treatments exposed to higher temperatures or extended durations converged into profiles characterized by pigment depletion. These trajectories confirm that anthocyanins and proanthocyanidins are highly susceptible to physicochemical instability, structural transformations, and oxidative losses upon prolonged water exposure [50,61,63]. Thus, time and temperature modulate individual metabolic pathways through distinct, non-linear mechanisms.

This dynamic behavior is further evidenced by the multivariate distinction between germinated flours and their corresponding water-soluble extracts. Germination modifies the structural accessibility and distribution of bioactive compounds without necessarily increasing their absolute concentration in every fraction [48,54,68]. Short-to-intermediate germination conditions specifically favored the aqueous recovery of flavonoids by altering cell wall interactions, whereas extended processing exacerbated thermal degradation [59,62]. This matrix remodeling also explains why antioxidant capacity (ORAC) decoupled from total phenolic concentrations. The total functional capacity reflects a complex synergy between phenolics and newly synthesized non-phenolic metabolites, such as GABA, γ-oryzanol, ascorbic acid, and bioactive peptides [68,72]. Therefore, optimizing black rice germination requires a multi-target approach: the functional consequences of the process must be evaluated in terms of matrix accessibility and phase distribution rather than solely maximizing individual bioactive classes.

Finally, certain methodological limitations must be considered when interpreting these findings. This study was conducted using a single black rice material, which may limit the direct extrapolation of the optimized conditions to cultivars with distinct initial phytochemical profiles. Additionally, the optimization focused strictly on germination time and temperature, while other processing factors potentially affecting bioactive retention were kept constant. The multi-response validation also demonstrated variable predictive accuracy among individual responses, indicating that the identified optimal condition should be regarded as an overall physiological compromise rather than an absolute maximum for every isolated parameter. Future research involving diverse pigmented cultivars, additional processing variables, and independent validation will help establish the broader technological applicability of these optimized conditions.

## 4. Materials and Methods

### 4.1. Plant Material

Black rice (*Oryza sativa* L.) seeds (cv. SCS120 Ônix) were received with the husk intact from the Santa Catarina Agricultural Research and Rural Extension Company (EPAGRI, Itajaí, Brazil). The seeds were dehulled using an MT2014 rice dehuller (Suzuki, São Paulo, Brazil), packed in biaxially oriented polypropylene bags, and stored at 4 °C under dark conditions in an industrial refrigerator (Pari, São Paulo, Brazil) until use. Access to the genetic material complied with Brazilian legislation on genetic heritage and associated traditional knowledge and was registered in the National System for the Management of Genetic Heritage and Associated Traditional Knowledge (SisGen), Ministry of the Environment, under registration number A880A53.

The reagents used in this study included sulfuric acid, sodium sulfate, copper sulfate, hydrochloric acid, boric acid, potassium chloride, methyl red, bromocresol green, sodium nitrate, aluminum chloride, monosodium phosphate monohydrate, and disodium phosphate heptahydrate were obtained from Synth (Diadema, SP, Brazil). Dansyl chloride, vanillin, Folin–Ciocalteu reagent (2 N), gallic acid, catechin, quercetin, 6-hydroxy-2,5,7,8-tetramethylchroman-2-carboxylic acid (Trolox), fluorescein, and 2,2′-azobis(2-amidinopropane) dihydrochloride (AAPH) were purchased from Sigma-Aldrich Co. (St. Louis, MO, USA). Methanol (HPLC grade), ethanol (HPLC grade), acetonitrile (HPLC grade), chloroform, γ-aminobutyric acid (GABA; purity >99%), and sodium acetate trihydrate were obtained from Merck KGaA (Darmstadt, Germany), while sodium carbonate, sodium hydroxide, and sodium hypochlorite were purchased from J.T. Baker (Phillipsburg, NJ, USA). Unless otherwise specified, all reagents were of analytical grade, and deionised water was used for the preparation of solutions and throughout the analytical procedures.

### 4.2. Proximate Composition

The proximate composition of black rice was determined by measuring moisture (method 934.01), protein (method 920.152; nitrogen-to-protein conversion factor, 5.95), and ash (method 942.05) according to the procedures of the Association of Official Analytical Chemists [73]. Lipid content was determined following the method described by [74], and total carbohydrate content was calculated by difference.

All analyses were performed in triplicate, and the results were expressed as a percentage (%).

### 4.3. Germination Procedure

**Figure 9:**
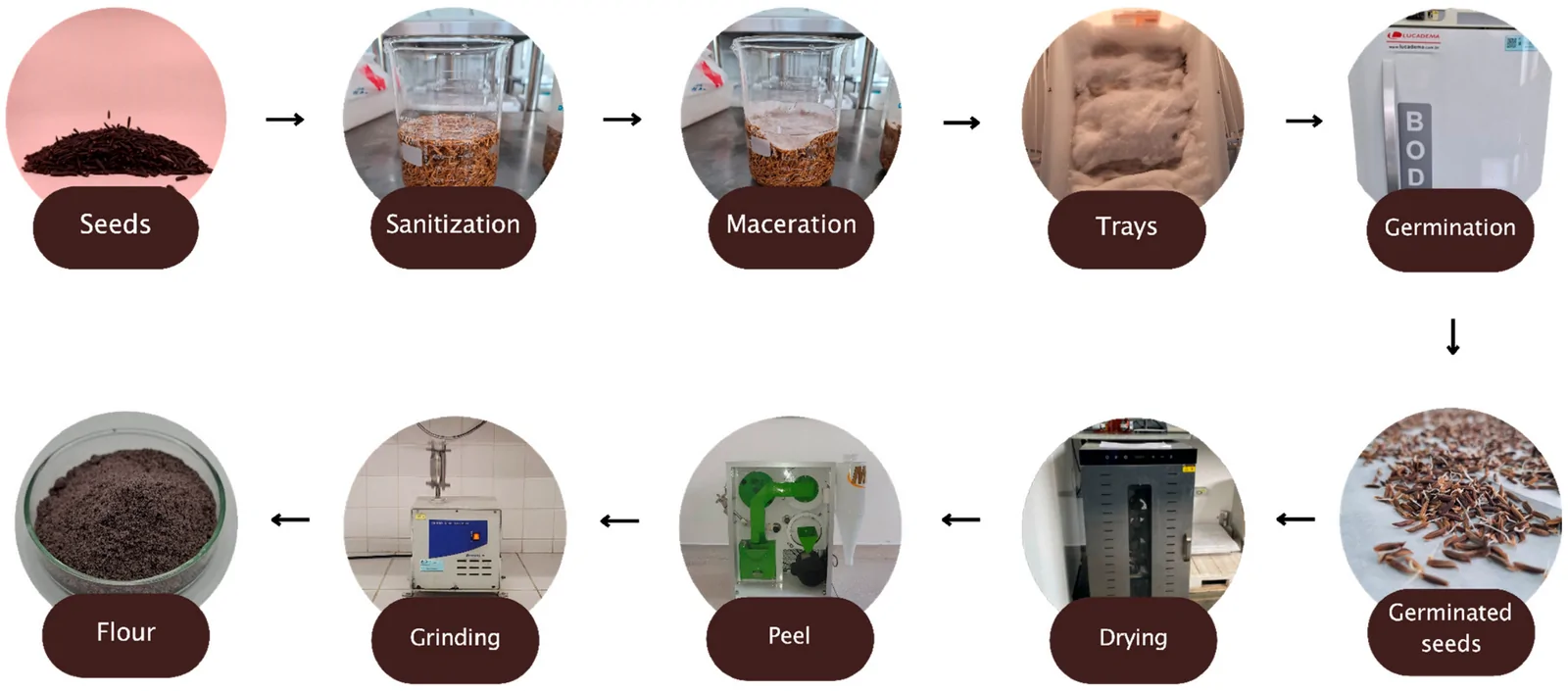
Schematic representation of the black rice germination process.

Germination was performed according to [75], with minor modifications. Intact black rice seeds (120 g) were sanitized in 200 ppm sodium hypochlorite solution for 30 min, rinsed thoroughly with distilled water, and soaked in distilled water (1:4, *w*/*v*) for 16 h at room temperature (approximately 20 °C), according to the water uptake curve. After soaking, the seeds were drained and distributed in polyethylene trays between layers of moist hydrophilic cotton separated by paper towels. Germination was carried out in a BOD germination chamber (RFE38, Lucadema, São José do Rio Preto, Brazil) under dark conditions. Relative humidity was maintained at 80–90% by periodically spraying the seeds with distilled water every 12 h and placing distilled water inside the chamber. At the end of germination, the seeds were dried in a food dehydrator (FRUGAL20, Alliere, Osasco, Brazil) at 45 °C for 12 h, cooled to room temperature, and manually separated from the radicles. The dried grains were subsequently dehulled and ground in a ball mill (TE-350, Tecnal, Piracicaba, Brazil) for 2 min to obtain flour with particle size smaller than 250 µm. The germinated flour was packaged in biaxially oriented polypropylene bags and stored at 4 °C under dark conditions until analysis. A schematic representation of the germination process is shown in Figure 9.

For comparative purposes, two reference samples were prepared. A non-germinated sample (NG) consisted of untreated black rice seeds and was used as the control. A soaked sample (SK) was prepared by subjecting black rice seeds to the same processing protocol as the germinated samples, except that the procedure was terminated after the soaking step, followed by drying under identical conditions. The inclusion of the SK sample al-lowed the effects of soaking to be distinguished from those specifically induced by germination, thereby enabling the identification of bioactive compounds affected during soaking before the onset of germination.

### 4.4. Experimental Design and Optimization

The germination experiment was designed using a Central Composite Design (CCD) to evaluate the effects of two independent variables: germination time (X_1_) and germination temperature (X_2_). The coded and actual levels of the experimental factors are presented in Table 4. Response Surface Methodology (RSM) was applied to model experimental responses and determine the optimal germination conditions. The experimental data were fitted to the second-order polynomial model proposed by [76], as described in Equation (10).

(10) $$Y=\beta_{0}+\beta_{1}x_{1}+\beta_{2}x_{2}+\beta_{11}x_{1}^{2}+\beta_{22}x_{2}^{2}+\beta_{12}x_{1}x_{2}+\varepsilon ,$$

where Y is the value of the dependent variable, β0 is the regression constant, βi, βii and βij are the linear, quadratic and interaction regression coefficients, respectively. While xi and xj are the coded values for the independent variables and ε is the experimental error.

The response variables included γ-aminobutyric acid (GABA) content, total phenolic compounds, total flavonoids, total anthocyanins, and oxygen radical absorbance capacity (ORAC), determined both in germinated black rice flour and in the corresponding water-soluble extracts. Multi-response optimization was performed using the desirability function proposed [77]. The independent variables were optimized within the experimental ranges evaluated, whereas all statistically significant response variables were set to be maximized. The relative importance assigned to each response ranged from 1 (lowest importance) to 5 (highest importance).

### 4.5. Evaluation of Germination Efficiency

GABA was determined according to the method described by [78], with minor modifications. Briefly, GABA was extracted from 1 g of sample using 75% (*v*/*v*) ethanol assisted by ultrasound using a CBU/100/3LDG ultrasonic bath (Planatec, São Paulo, Brazil, 40 kHz, 100 W) for 10 min. After centrifugation in a 206BL centrifuge (Fanem, Guarulhos, Brazil) (3200× *g*, 5 min), the extraction was repeated, and the combined extracts were adjusted to a final volume of 10 mL. GABA was derivatized with dansyl chloride before chromatographic analysis by mixing the extract with 10 mM dansyl chloride in acetonitrile and 5 M sodium carbonate buffer (pH 10), followed by ultrasonication, incubation at 65 °C for 25 min, and centrifugation (9600× *g*, 15 min, 4 °C) using a refrigerated centrifuge (SL-5GR, Spinlab, Ribeirão Preto, Brazil). Quantification was performed using an Agilent 1260 Infinity HPLC system (Agilent Technologies, Santa Clara, CA, USA) equipped with a diode-array detector (DAD) and a Zorbax Eclipse C18 column (4.6 × 100 mm, 3.5 μm). Separation was achieved using a gradient of 0.05 M sodium phosphate buffer (pH 7.2) and acetonitrile at a flow rate of 1.0 mL min^−1^. Detection was carried out at 254 nm with an injection volume of 10 μL. Quantification was based on an external calibration curve prepared from γ-aminobutyric acid standard (Merck, Darmstadt, Germany, >99% purity; 0–184 mg L^−1^; r = 0.9994), and the results were expressed as mg GABA per 100 g dry basis (mg 100 g^−1^ db).

### 4.6. Preparation of Water-Soluble Extracts

Water-soluble extracts were prepared from germinated black rice flour and the non-germinated control. Briefly, 25 g (dry basis) of flour were mixed with potable water (1:6, *w*/*v*) and subjected to a static mashing process in a water bath (157/22, Marconi, São José do Rio Preto, Brazil) according to the temperature profile described by [78]. After mashing, the samples were cooled, homogenized using an Ultra-Turrax homogenizer (TE-102, Tecnal, Piracicaba, Brazil) for 2 min at 1300 rpm, and filtered through a 0.88 mm mesh. The final volume was adjusted to 150 mL with potable water. The extracts were transferred to polypropylene containers and stored at −18 °C in a freezer (RCFB32, Canoas, Rio Grande do Sul, Brazil) under dark conditions until analysis.

**Figure 10:**
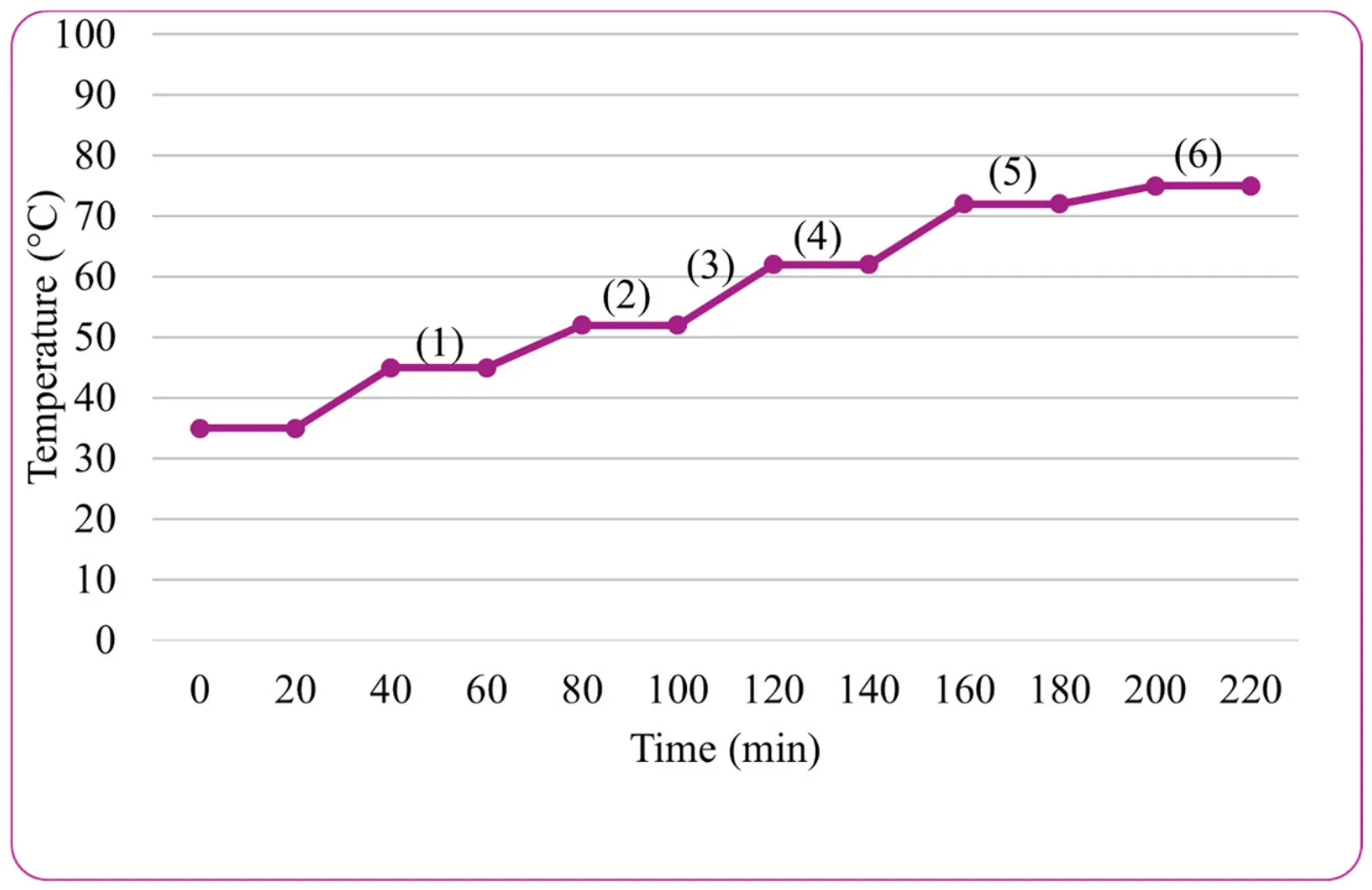
Mashing temperature profile used to produce black rice water-soluble extracts and the corresponding enzymatic activities. (1) Optimal temperature for the action of hemicellulases (40–45 °C) and exopeptidases (40–50 °C). (2) Optimal temperature for the action of endopeptidases (50–60 °C). (3) The temperature range that includes the ramp favors the activity of dextrinases (55–60 °C). (4) Optimal temperature for the action of *β*-amylases (60–65 °C). (5) Optimal temperature for the action of *α*-amylases (70–75 °C). (6) Enzymatic inactivation (75 °C).

**Figure 11:**
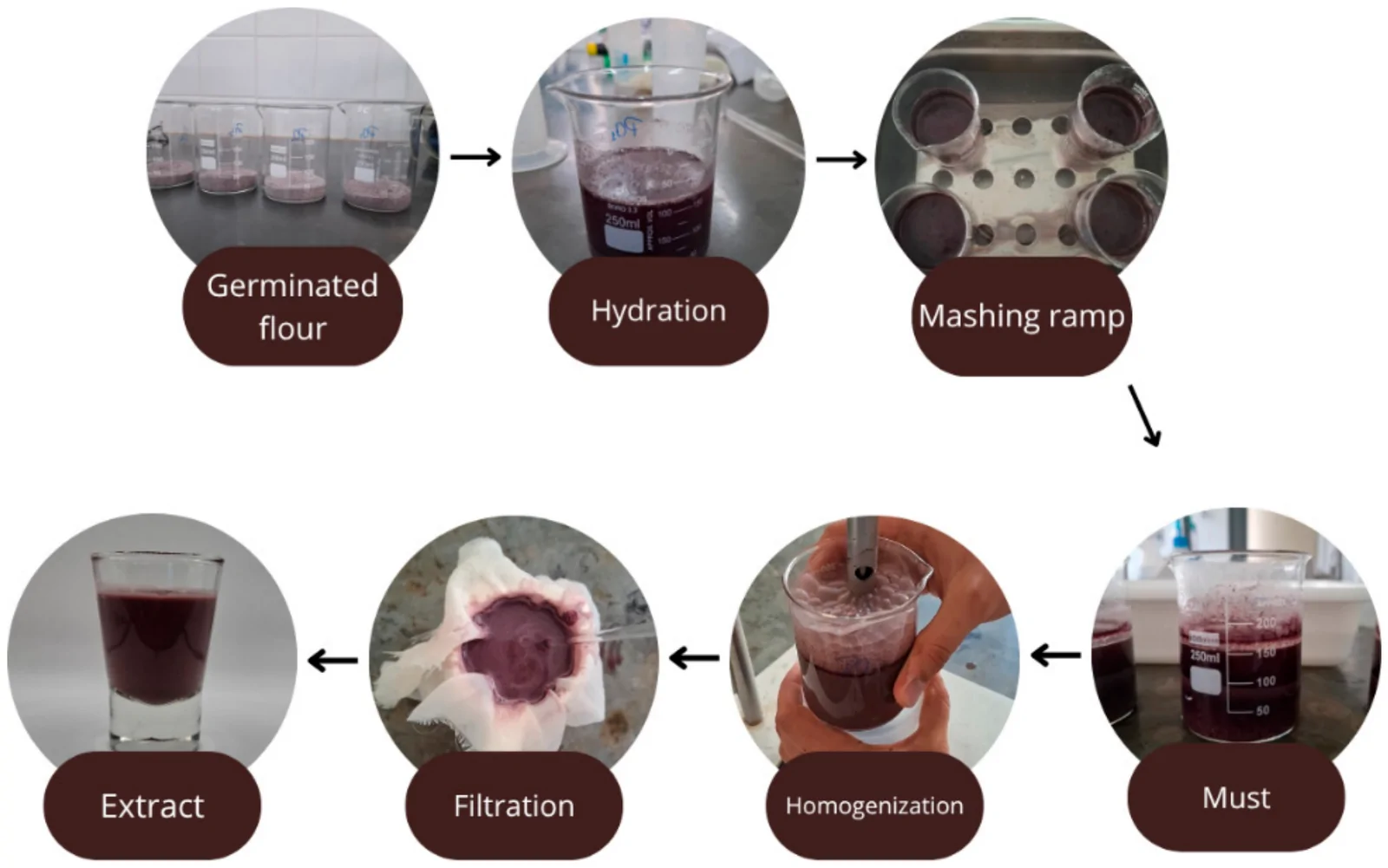
Workflow for the preparation of black rice water-soluble extracts.

The mashing temperature profile and the corresponding enzymatic activity are illustrated in Figure 10, whereas the overall workflow for water-soluble extract production is presented in Figure 11.

### 4.7. Determination of Bioactive Compounds and Antioxidant Capacity

#### 4.7.1. Preparation of Phenolic Extracts

Phenolic extracts were prepared from the control, soaked, and germinated black rice flours according to [79], with minor modifications. Briefly, 0.5 g of flour was mixed with 5 mL of 70% (*v*/*v*) methanol in a 15 mL Falcon tube, vortex-mixed for 1 min, sonicated in an ultrasonic bath (CBU/100/3LDG, Planatec, São Paulo, Brazil; 40 kHz, 100 W) for 5 min, and centrifuged at 3200× *g* for 5 min using a Sorvall ST8 centrifuge (Thermo Fisher Scientific, Waltham, MA, USA). The supernatant was collected, adjusted to 10 mL with extraction solvent, and stored at −18 °C until analysis. Before analysis, aliquots of the extracts were centrifuged at 9600× *g* for 10 min at 20 °C using a refrigerated centrifuge (SL-5GR, Spinlab, Ribeirão Preto, Brazil), freeze-dried for 48 h (Alpha 2–4 Lplus, Christ, São Paulo, Brazil), reconstituted in 70% (*v*/*v*) methanol, sonicated for 10 min, and centrifuged again under the same conditions. This procedure was adopted to ensure complete solubilization of catechin and quercetin standards and to maintain solvent compatibility between samples and calibration standards. The resulting extracts were used for the determination of total soluble phenolic compounds, total flavonoids, proanthocyanidins, anthocyanins, and antioxidant capacity.

#### 4.7.2. Preparation of Phenolic Extracts

Total soluble phenolic compounds (TSPC) were determined using the Folin–Ciocalteu method according to [80]. Briefly, 100 μL of extract was mixed with 250 μL of 0.2 N Folin–Ciocalteu reagent, 3 mL of distilled water, and 1 mL of 15% (*w*/*v*) sodium carbonate. After homogenization, the reaction mixtures were incubated in the dark for 30 min before absorbance measurement.

For flour samples, absorbance was measured at 750 nm using a Biochrom UVM340 microplate spectrophotometer (Asys, Cambridge, UK), and quantification was performed using a gallic acid calibration curve (0–350 mg L^−1^; r = 0.9913). Results were expressed as mg gallic acid equivalents per 100 g dry basis (mg GAE 100 g^−1^ d.b.).

For water-soluble extracts, absorbance was measured at 750 nm using a SpectraMax^®^ Paradigm^®^ microplate reader (Molecular Devices, San Jose, CA, USA). Quantification was based on a gallic acid calibration curve (0–600 mg L^−1^; r = 0.983), and the results were expressed as mg gallic acid equivalents per 100 mL of extract (mg GAE 100 mL^−1^). All analyses were performed in triplicate.

#### 4.7.3. Total Flavonoids Content

Total flavonoid content (TFC) was determined according to the colorimetric method described by [81], with minor modifications. The assay was performed independently for flour samples and water-soluble extracts. Briefly, 60 μL of extract was mixed with 440 μL of ultrapure water, followed by the sequential addition of 32 μL of 5% (*w*/*v*) sodium nitrite and 32 μL of 10% (*w*/*v*) aluminum chloride, with reaction times of 5 and 6 min, respectively. Subsequently, 200 μL of 10% (*w*/*v*) sodium hydroxide and 210 μL of ultrapure water were added, and the mixtures were homogenized before absorbance measurement.

For flour samples, absorbance was measured at 510 nm using a Biochrom UVM340 microplate spectrophotometer (Asys, Cambridge, UK). Quantification was performed using a quercetin calibration curve (0–600 mg L^−1^; *r* = 0.990), and the results were expressed as mg quercetin equivalents per 100 g dry basis (mg QE 100 g^−1^ db).

For water-soluble extracts, absorbance was measured at 510 nm using a SpectraMax^®^ Paradigm^®^ microplate reader (Molecular Devices, San Jose, CA, USA). Quantification was based on a quercetin calibration curve (0–600 mg L^−1^; *r* = 0.981), and the results were expressed as mg quercetin equivalents per 100 mL of extract (mg QE 100 mL^−1^). All analyses were performed in triplicate.

#### 4.7.4. Total Anthocyanins Content

Total anthocyanin content (TAC) was determined according to AOAC Official Method 2005.02 [73], with minor modifications. Flour samples and water-soluble extracts were diluted in 0.025 M potassium chloride buffer (pH 1.0) and the corresponding buffer required by the pH differential method. Absorbance was measured using a UV–Vis spectrophotometer (UV-M51, Bel Photonics, Monza, Italy), and anthocyanin concentration was calculated according to Equation (11) and expressed as cyanidin-3-O-glucoside equivalents. Results were expressed as mg cyanidin-3-glucoside equivalents per 100 g dry basis (mg C3G 100 g^−1^ d.b.) for flour samples and mg cyanidin-3-glucoside equivalents per 100 mL of water-soluble extract (mg C3G 100 mL^−1^) for the extracts. All analyses were performed in triplicate.

(11) $$\mathrm{TAC}=(A\times 449.2\;{g.mol}^{-1}\times d\times 10^{3})/26,900,$$

where TAC—total anthocyanin content; A = Absorbance at 520 nm; d = dilution; 449.2 = molar mass of cyanidin-3-O-glucoside; 26,900 = molar extinction coefficient of cyanidin-3-O-glucoside·cm^−1^.

#### 4.7.5. Total Proanthocyanidins Content

Total proanthocyanidin content (TPAC) was determined according to the vanillin assay described by [79], with minor modifications. The analysis was performed independently for flour samples and water-soluble extracts. Briefly, 100 μL of extract was mixed with 600 μL of 4% (*w*/*v*) vanillin solution prepared in methanol and 300 μL of hydrochloric acid (HCl, analytical grade). After homogenization, the reaction mixtures were incubated for color development before absorbance measurement.

For flour samples, absorbance was measured at 500 nm using a Biochrom UVM340 microplate spectrophotometer (Asys, Cambridge, UK). Quantification was performed using a catechin calibration curve (0–600 mg L^−1^; r = 0.990), and the results were expressed as mg catechin equivalents per 100 g dry basis (mg CE 100 g^−1^ db).

For water-soluble extracts, absorbance was measured at 500 nm using a SpectraMax^®^ Paradigm^®^ microplate reader (Molecular Devices, San Jose, CA, USA). Quantification was based on a catechin calibration curve (0–600 mg L^−1^; r = 0.997), and the results were expressed as mg catechin equivalents per 100 mL of extract (mg CE 100 mL^−1^). All analyses were performed in triplicate.

#### 4.7.6. Antioxidant Capacity

The antioxidant capacity of methanolic extracts obtained from the control, soaked, and germinated black rice flours, as well as the water-soluble extracts, was determined using the oxygen radical absorbance capacity (ORAC) assay according to [82], with the modifications proposed by [83]. Briefly, 20 μL of sample or Trolox standard solution (0–0.75 mM) was mixed with 120 μL of 70 nM fluorescein in black 96-well microplates and incubated at 37 °C for 15 min. The reaction was initiated by adding 200 μL of 12 mM 2,2′-azobis(2-methylpropionamidine) dihydrochloride (AAPH). Fluorescence decay was monitored every 1 min for 80 min using a SpectraMax^®^ Paradigm^®^ microplate reader (Molecular Devices, San Jose, CA, USA) at an excitation wavelength of 485 nm and an emission wavelength of 525 nm. The microplates were maintained at 37 °C with orbital shaking for 5 s before each reading.

Antioxidant capacity was calculated from the area under the fluorescence decay curve using Trolox as the reference standard. Results were expressed as mM Trolox equivalents per 100 g dry basis (mM TE 100 g^−1^ db) for flour samples and μM Trolox equivalents per 100 mL of water-soluble extract (μM TE 100 mL^−1^) for the extracts.

### 4.8. Statistical Analysis

Experimental data were analyzed using Response Surface Methodology (RSM) to fit second-order polynomial models, using a Statistica 7.0 software (StatSoft Inc., Tulsa, OK, USA). Regression coefficients were estimated, and the significance of the models and individual terms was evaluated by analysis of variance (ANOVA) at a 10% significance level (*p* < 0.10). Models presenting a coefficient of determination (R^2^) ≥ 0.80 were considered suitable for response prediction. For the response surface analysis, analysis of variance (ANOVA) was performed considering a significance level of α = 0.10 for screening the effects of germination time and temperature. This threshold was adopted considering the intrinsic biological variability associated with seed germination and to reduce the risk of excluding potentially relevant process effects of moderate magnitude within the experimental domain. Model adequacy was not assessed solely based on the coefficient of determination (R^2^). The overall significance of the regression model, adjusted R^2^, predicted R^2^, and lack-of-fit test were also considered to evaluate goodness of fit and predictive performance. Models showing adequate overall statistical significance and satisfactory goodness-of-fit and predictive parameters were retained for response-surface interpretation and multi-response optimization, whereas non-significant models were used only for descriptive interpretation of the experimental data. Multivariate analysis was performed using MetaboAnalyst 6.0 (Xia Lab, Montreal, QC, Canada). Experimental data were log_10_-transformed and auto-scaled before analysis. Relationships among samples and response variables were investigated by principal component analysis (PCA), partial least squares discriminant analysis (PLS-DA), and hierarchical clustering analysis (heatmap).

### 4.9. AI Use Statement

Generative artificial intelligence (GenAI), through ChatGPT 5.5 (OpenAI, San Francisco, CA, USA), was used as a supporting tool for language revision, grammatical correction, improvement of textual clarity, and organization of selected sections of the manuscript. All AI-assisted content was critically reviewed, edited, and validated by the authors. The scientific content, study conceptualization, methodological decisions, experimental design and execution, data analysis and interpretation, and conclusions presented in this study are entirely the responsibility of the authors. GenAI was not used as a bibliographic source, for generating or analyzing experimental data, or as a substitute for critical scientific analysis or human authorship. The authors take full responsibility for the accuracy, integrity, originality, and scientific content of the manuscript and graphical abstract.

## 5. Conclusions

This study demonstrated that controlled germination is a promising biotechnological strategy for modulating the bioactive profile and functional properties of black rice flour and its water-soluble extracts. Germination time and temperature differentially affected GABA accumulation, phenolic compounds, flavonoids, anthocyanins, proanthocyanidins, and antioxidant capacity, indicating that the response of black rice to germination involves simultaneous changes in metabolite accumulation, transformation, retention, and extractability.

GABA, which was not detected in the untreated and soaked samples, accumulated markedly after germination, while the recovery of soluble phenolic compounds, flavonoids, and antioxidant constituents increased in the aqueous extracts. In contrast, anthocyanins and proanthocyanidins were generally more susceptible to losses during soaking and prolonged germination. These findings highlight that germination does not uniformly increase all bioactive constituents but rather modifies their distribution and accessibility within the grain matrix and between solid and aqueous fractions.

Multivariate analysis confirmed the formation of distinct biochemical profiles according to the germination conditions, whereas multi-response optimization identified 27 h and 19.7 °C as the most suitable processing conditions, with an overall desirability of 70.81%. These conditions provided an appropriate compromise between GABA accumulation, retention of bioactive compounds in the flour, and antioxidant capacity of the water-soluble extracts. Therefore, controlled germination represents a potential approach for obtaining black rice ingredients with tailored functional profiles for applications in functional foods, plant-based beverages, and other value-added products. Nevertheless, the mechanistic interpretations proposed in this study should be considered plausible explanations supported by the literature, since enzyme activities, gene expression, metabolic fluxes, and individual metabolite profiles were not directly evaluated. Further research should therefore investigate the stability of the bioactive compounds during food processing and storage, characterize individual phenolic and non-phenolic constituents, and assess their gastrointestinal bioaccessibility and in vivo biological effects.

## Figures and Tables

**Table 1:** Effect of germination conditions on GABA accumulation in black rice flour.

Trials	mg GABA·100 g^−1^ Flour (d.b.)
NG	n.d.
1	18.19 ± 0.02
2	15.91 ± 0.46
3	12.58 ± 0.33
4	8.69 ± 0.13
5	12.32 ± 0.05
6	8.01 ± 0.10
7	21.02 ± 0.34
8	14.26 ± 0.14
9	13.04 ± 0.81
10	11.09 ± 0.32
11	13.77 ± 0.05
12	11.58 ± 0.84
SK	n.d.
*p*-value	<0.001
R^2^	0.9556
R^2^-Adjusted	0.9302
Lack of fit	0.850

Values are expressed as mean ± standard deviation (n = 3). NG, non-germinated black rice flour (control); SK, soaked black rice flour; GABA, γ-aminobutyric acid; n.d., not detected; d.b., dry basis.

**Table 2:** Bioactive compounds and antioxidant capacity of black rice flours and their corresponding soluble extracts obtained under different germination conditions.

Trial	TSPC		TFC		TAC		TPAC		ORAC	
	Flour (mg GAE·100 g^−1^, d.b.)	Soluble Extract (mg GAE·100 mL^−1^)	Flour (mg QE·100 g^−1^, d.b.)	Soluble Extract (mg QE·100 mL^−1^)	Flour (mg C3G·100 g^−1^, d.b.)	Soluble Extract (mg C3G·100 mL^−1^)	Flour (mg CE·100 g^−1^, d.b.)	Soluble Extract (mg CE·100 mL^−1^)	Flour (µM TE·100 g^−1^, d.b.)	Soluble Extract (µM TE·100 mL^−1^)
NG	1172.93 ± 38.13	154.24 ± 21.42	672.02 ± 11.33	302.89 ± 25.46	6250.44 ± 278.58	339.63 ± 18.55	408.13 ± 24.25	63.27 ± 3.98	22.97 ± 0.01	2851.54 ± 240.35
1	1201.75 ± 106.93	132.21 ± 7.29	716.74 ± 17.84	413.41 ± 26.33	4642.81 ± 49.56	328.28 ± 5.97	250.73 ± 7.07	37.68 ± 2.10	28.37 ± 1.81	2832.80 ± 227.47
2	1294.51 ± 3.10	136.78 ± 5.30	573.67 ± 13.34	310.28 ± 26.89	4207.63 ± 118.57	287.84 ± 4.40	234.29 ± 11.44	34.53 ± 2.96	29.30 ± 0.39	2787.26 ± 59.67
3	1140.44 ± 80.22	143.95 ± 10.71	816.22 ± 12.84	362.29 ± 18.32	4436.02 ± 308.54	370.95 ± 13.60	211.14 ± 2.87	45.53 ± 2.31	27.57 ± 1.14	2783.76 ± 58.42
4	1196.50 ± 126.73	148.00 ± 7.31	375.02 ± 9.49	249.83 ± 17.88	3178.54 ± 266.05	226.72 ± 7.12	139.57 ± 5.79	13.51 ± 2.83	28.10 ± 1.58	3306.57 ± 192.57
5	1059.01 ± 46.43	140.54 ± 7.14	834.18 ± 26.56	360.11 ± 18.89	4538.73 ± 358.61	373.04 ± 11.33	304.17 ± 10.65	59.05 ± 4.17	27.77 ± 0.52	2740.52 ± 206.01
6	1165.85 ± 83.70	129.36 ± 7.68	534.27 ± 19.60	273.09 ± 18.81	3568.45 ± 84.63	284.43 ± 3.71	192.84 ± 6.48	36.64 ± 1.89	28.08 ± 0.32	3010.80 ± 209.71
7	1152.27 ± 53.93	138.53 ± 9.79	729.72 ± 20.05	362.68 ± 21.50	4440.38 ± 112.38	362.68 ± 11.50	253.19 ± 9.50	51.40 ± 8.99	28.19 ± 2.40	2543.56 ± 215.07
8	1172.49 ± 31.58	148.27 ± 8.80	410.25 ± 17.00	290.22 ± 10.84	3415.91 ± 134.07	354.45 ± 11.36	165.72 ± 3.58	30.99 ± 3.02	25.66 ± 0.09	3030.23 ± 139.16
9	1193.66 ± 54.78	148.58 ± 5.73	479.06 ± 12.16	320.39 ± 21.13	4234.64 ± 212.51	329.95 ± 0.55	231.05 ± 3.92	50.47 ± 3.92	25.24 ± 0.34	2185.26 ± 169.56
10	1123.25 ± 20.43	148.47 ± 10.43	543.39 ± 9.05	322.12 ± 21.48	4189.67 ± 271.83	349.63 ± 5.00	220.05 ± 17.27	57.50 ± 5.09	25.38 ± 1.13	2290.83 ± 173.91
11	1160.15 ± 30.09	148.32 ± 10.02	607.14 ± 14.11	321.23 ± 20.19	3972.64 ± 188.50	335.24 ± 13.92	225.55 ± 18.35	53.99 ± 4.50	25.49 ± 0.98	2201.76 ± 139.78
12	1117.22 ± 29.69	149.37 ± 5.85	432.84 ± 8.10	326.03 ± 25.39	4099.26 ± 198.00	343.96 ± 52.98	228.30 ± 18.30	52.23 ± 3.04	27.68 ± 2.03	2444.30 ± 74.54
SK	1155.56 ± 47.36	-	471.26 ± 12.51	-	4692.53 ± 102.54	-	226.84 ± 10.83	-	-	-
*p*-value	0.418	0.007	0.005	<0.001	<0.001	0.008	0.003	0.026	0.418	0.001
R^2^	0.4948	0.8393	0.8494	0.9346	0.9497	0.8866	0.9247	0.8316	0.7163	0.9515
R^2^-adjusted	0.0738	0.7476	0.7633	0.9101	0.9308	0.7921	0.8619	0.6912	0.4798	0.9115
Lack of fit	0.151	0.161	0.523	0.125	0.476	0.533	0.156	0.388	0.703	0.669

Values are expressed as mean ± standard deviation (n = 3). TSPC, total soluble phenolic compounds (mg GAE·100 g^−1^ for flour and mg GAE·100 mL^−1^ for soluble extracts); TFC, total flavonoid content (mg QE·100 g^−1^ for flour and mg QE·100 mL^−1^ for soluble extracts); TAC, total anthocyanin content (mg C3G·100 g^−1^ for flour and mg C3G·100 mL^−1^ for soluble extracts); TPAC, total proanthocyanidin content (mg CE·100 g^−1^ for flour and mg CE·100 mL^−1^ for soluble extracts); ORAC, oxygen radical absorbance capacity (µmol TE·100 g^−1^ for flour and µmol TE·100 mL^−1^ for soluble extracts). GAE, gallic acid equivalents; QE, quercetin equivalents; C3G, cyanidin-3-glucoside equivalents; TE, Trolox equivalents; CE, catechin equivalent; d.b., dry basis; R^2^ = Regression Determination.

**Table 3:** Predicted and experimental responses, relative deviation, and validation of the multi-response optimization model for germinated black rice.

Independent Variables					
Parameters	Goal	Importance	Solution		
			Coded Value	Real Value	
Time (h)	In range	3	−1.29	27.06	
Temperature (°C)	In range	3	−0.51	19.7	
Dependent Variables					
Parameters	Goal	Importance	Solution	Optimal Point Values	Relative Deviation (%)
GABA content in flour	maximize	5	14.61	14.67 ± 0.46	0.41
TSPC in the extract	maximize	5	135.86	175.76 ± 11.11	29.37
TFC in flour	maximize	5	813.97	1176.49 ± 83.92	44.54
TFC in the extract	maximize	5	392.30	412.79 ± 33.03	5.22
TAC in flour	maximize	5	4607.67	3860.4 ± 102.11	−16.22
TAC in the extract	maximize	5	362.73	376.98 ± 4.36	3.93
TPAC in flour	maximize	5	279.94	263.2 ± 21.76	−5.98
TPAC in the extract	maximize	5	50.23	48.47 ± 6.14	−3.50
ORAC of the extract	maximize	5	2770.71	3712.11 ± 380.42	33.98
Desirability 70.81%					

GABA, γ-aminobutyric acid (mg GABA 100 g^−1^ dry basis (d.b.)); TSPC, total soluble phenolic compounds (mg GAE 100 g^−1^ d.b. for flour and mg GAE 100 mL^−1^ extract); TFC, total flavonoid content (mg quercetin equivalents (QE) 100 g^−1^ d.b. for flour and mg QE 100 mL^−1^ extract); TAC, total anthocyanin content (mg cyanidin-3-glucoside equivalents (C3GE) 100 g^−1^ d.b. for flour and mg C3GE 100 mL^−1^ extract); TPAC, total proanthocyanidin content (mg catechin equivalents (CE) 100 g^−1^ d.b. for flour and mg CE 100 mL^−1^ extract); ORAC, oxygen radical absorbance capacity (µM Trolox 100 mL^−1^ extract). Relative deviation (%) was calculated as ((Predicted − Experimental)/Predicted) × 100.

**Table 4:** Coded and actual levels for evaluating the time and temperature performance of black rice germination.

Trial	Coded Levels		Real Levels	
	x_1_	x_2_	X_1_	X_2_
1	−1	−1	34.5	16.6
2	1	−1	85.5	16.6
3	−1	1	34.5	29.4
4	1	1	85.5	29.4
5	−1.41	0	24.0	23.0
6	1.41	0	96.0	23.0
7	0	−1.41	60.0	14.0
8	0	1.41	60.0	32.0
9	0	0	60.0	23.0
10	0	0	60.0	23.0
11	0	0	60.0	23.0
12	0	0	60.0	23.0

where x_1_ and X_1_ denote the coded and actual values, respectively, of germination time, whereas x_2_ and X_2_ denote the coded and actual values, respectively, of germination temperature.

## Data Availability

The original contributions presented in this study are included in the article. Further inquiries can be directed to the corresponding author.
